# Aberrant expression of RSK1 characterizes high‐grade gliomas with immune infiltration

**DOI:** 10.1002/1878-0261.12595

**Published:** 2019-12-11

**Authors:** Glaucia N. M. Hajj, Fernanda F. da Silva, Bárbara de Bellis, Fernanda C. S. Lupinacci, Hermano M. Bellato, Juvanier R. Cruz, Claudionor N. C. Segundo, Igor V. Faquini, Leuridan C. Torres, Paulo I. Sanematsu, Maria D. Begnami, Vilma R. Martins, Martín Roffé

**Affiliations:** ^1^ International Research Center A.C.Camargo Cancer Center São Paulo Brazil; ^2^ National Institute of Science and Technology in Oncogenomics São Paulo Brazil; ^3^ Department of Clinical Oncology Hospital do Câncer de Pernambuco Recife Brazil; ^4^ Department of Neurosurgery Hospital da Restauração Recife Brazil; ^5^ Translational Research Laboratory Prof. C. A. Hart Instituto de Medicina Integral Prof. Fernando Figueira Recife Brazil; ^6^ Department of Neurosurgery A.C.Camargo Cancer Center São Paulo Brazil; ^7^ Department of Pathology A.C.Camargo Cancer Center São Paulo Brazil

**Keywords:** gene signature, glioma, immune infiltrate, LAPTM5, mesenchymal subtype, RSK

## Abstract

The p90 ribosomal S6 kinase (RSK) family, a downstream target of Ras/extracellular signal‐regulated kinase signaling, can mediate cross‐talk with the mammalian target of rapamycin complex 1 pathway. As RSK connects two oncogenic pathways in gliomas, we investigated the protein levels of the RSK isoforms RSK1–4 in nontumoral brain (NB) and grade I‐IV gliomas. When compared to NB or low‐grade gliomas (LGG), a group of glioblastomas (GBMs) that excluded long‐survivor cases expressed higher levels of RSK1 (RSK1^hi^). No difference was observed in RSK2 median‐expression levels among NB and gliomas; however, high levels of RSK2 in GBM (RSK2^hi^) were associated with worse survival. RSK4 expression was not detected in any brain tissues, whereas RSK3 expression was very low, with GBM demonstrating the lowest RSK3 protein levels. RSK1^hi^ and, to a lesser extent, RSK2^hi^ GBMs showed higher levels of phosphorylated RSK, which reveals RSK activation. Transcriptome analysis indicated that most RSK1^hi^ GBMs belonged to the mesenchymal subtype, and RSK1 expression strongly correlated with gene expression signature of immune infiltrates, in particular of activated natural killer cells and M2 macrophages. In an independent cohort, we confirmed that RSK1^hi^ GBMs exclude long survivors, and RSK1 expression was associated with high protein levels of the mesenchymal subtype marker lysosomal protein transmembrane 5, as well as with high expression of CD68, which indicated the presence of infiltrating immune cells. An RSK1 signature was obtained based on differentially expressed mRNAs and validated in public glioma datasets. Enrichment of RSK1 signature followed glioma progression, recapitulating RSK1 protein expression, and was associated with worse survival not only in GBM but also in LGG. In conclusion, both RSK1 and RSK2 associate with glioma malignity, but displaying isoform‐specific peculiarities. The progression‐dependent expression and association with immune infiltration suggest RSK1 as a potential progression marker and therapeutic target for gliomas.

## Graphical abstract

RSK1 protein levels are higher in a subpopulation of glioblastomas (RSK1^hi^ GBMs) than in low‐grade gliomas and also after recurrence. Long‐survivor cases are excluded from RSK1^hi^ GBMs. Transcriptome analysis of RSK1^hi^ GBMs indicates the presence of immune infiltrate. An RSK1 gene signature recapitulates the increase in RSK1 levels in higher‐grade gliomas, and its enrichment is associated with poor survival.

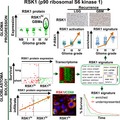

**Abbreviations**
ACCCC: A.C.Camargo Cancer Center
CL: classical
DEG: differentially expressed gene
ERK: extracellular signal‐regulated kinase
FFPE: formalin‐fixed, paraffin‐embedded
GBM: glioblastoma
G‐CIMP: glioma CpG island methylator phenotype
GSVA: Gene set variation analysis
HCP: Hospital do Cancer de Pernambuco
HR: Hospital da Restauração
IDH: isocitrate dehydrogenase
IHC: immunohistochemistry
LAPTM5: lysosomal protein transmembrane 5
LGG: low‐grade glioma
ME: mesenchymal
mTORC1: mammalian target of rapamycin complex 1
NB: nontumoral brain
NK: natural killer cells
NL: neural
PN: proneural
RPPA: reverse‐phase protein array
RSK: p90 ribosomal S6 kinase
TCGA: The Cancer Genome Atlas
TMA: tissue microarray

## 1 Introduction

Gliomas are tumors of the central nervous system resulting from the malignant transformation of glial cells, their intermediate precursors, and neural stem cells (Huse and Holland, 2010; Zong *et al.*, 2015). Gliomas are classified into different grades (I–IV) according to their histological characteristics (Louis *et al.*, 2016). High‐grade gliomas, III and IV, are considered malignant (Lienhart *et al.*, 2008). The incidence of malignant gliomas is 17 000 new cases per year (Omuro and DeAngelis, 2013). Astrocytomas are the most common type of gliomas. In particular, grade IV astrocytoma, also called glioblastoma (GBM, formerly called GBM multiforme), represents 82% of malignant gliomas. GBM is highly lethal, with the average patient surviving only 12–15 months. Standard treatment involves surgical resection when possible, but because of its infiltrative nature, GBMs cannot be completely removed. Therefore, adjuvant treatment, consisting of radiotherapy and chemotherapy with the alkylating agent temozolomide, is performed after surgery. Nevertheless, almost all GBMs eventually recur and the mean disease progression time after treatment is 6.9 months (Omuro and DeAngelis, 2013).

The Cancer Genome Atlas (TCGA) and other similar initiatives applied high‐throughput genomic techniques to hundreds of GBMs and low‐grade gliomas (LGG; grades II and III) (Ceccarelli *et al.*, 2016). These efforts resulted in numerous molecular classifications of gliomas. For GBMs, TCGA proposed an initial classification into four molecular subtypes (classical, mesenchymal, proneural, and neural) based on transcriptome data (Verhaak *et al.*, 2010). The isocitrate dehydrogenase gene 1 and 2 (IDH1/IDH2) mutational status and the hypermethylator phenotype [glioma CpG island methylator phenotype (G‐CIMP)], both characteristic of secondary GBMs and associated with longer survival times, were then considered to improve the TCGA classification (Noushmehr *et al.*, 2010; Turkalp *et al.*, 2014). Analysis of GBMs together with LGG essentially divided gliomas into IDH mutant (consisting of most LGG and secondary GBMs) and IDH wild‐type (mostly primary GBMs) (Ceccarelli *et al.*, 2016). Later on, TCGA eliminated the neural subtype classification for GBMs. Despite all these efforts, only a limited amount of the generated information was translated to patients. A few prognostic markers are considered for GBM, including IDH1 mutation status (Turkalp *et al.*, 2014).

It was observed that 88% of GBMs show alterations in at least one of the major components of the receptor tyrosine kinase/phosphatidylinositol‐3 kinase/protein kinase B/mammalian target of rapamycin complex 1 (mTORC1) signaling pathways (Akhavan *et al.*, 2010). Ras/extracellular signal‐regulated kinase (ERK) signaling is also frequently altered in GBMs, and one of the main causes is the loss of neurofibromin 1 in 18% of patients (Akhavan *et al.*, 2010). A direct target of ERK signaling is the p90 kDa ribosomal protein S6 kinase family (RSK). In humans, there are four isoforms of RSK with a high degree of homology (RSK1–4) (Anjum and Blenis, 2008). RSKs can phosphorylate various substrates, and it has been suggested that RSKs mediate cross‐talk between the Ras/ERK and mTORC1 pathways. RSKs are known to be important in several cancers, controlling processes such as proliferation, mRNA translation, and survival (Houles and Roux, 2018). Despite its strategic position between two important oncogenic pathways in GBMs, very little is known about the role of RSK in gliomas. It was reported that mesenchymal subtype GBMs showed a marginal increase in phosphorylated RSK1 (T359/S363) relative to proneural GBMs (Brennan *et al.*, 2013). RSK2 is thought to be a target of miR‐218 and a regulator of GBM migration and invasion (Mathew *et al.*, 2015; Sulzmaier *et al.*, 2016). However, the last article failed to exclude any involvement of the other RSK isoforms. Moreover, the use of a nonspecific RSK inhibitor (BI‐D1870 (Roffé *et al.*, 2015)) could mislead the correct interpretation of RSK2 involvement (Sulzmaier *et al.*, 2016). Considering the potential of RSKs as important modulators of oncogenic pathways, we aimed to comprehensively characterize the expression of RSK family isoforms in gliomas and underscore its implications.

## 2 Materials and methods

### 2.1 Glioma patient cohorts

Formalin‐fixed paraffin‐embedded (FFPE) samples of astrocytomas of different grades (I–IV) treated at the A.C.Camargo Cancer Center (ACCCC), São Paulo, Brazil, from 1980 to 2004 were used for the construction of tissue microarrays (TMA) (Alvarenga *et al.*, 2017; Machado *et al.*, 2018): I—pilocytic (*N* = 36), II—diffuse (*N* = 40), III—anaplastic (*N* = 14), and IV—GBM (*N* = 85). The TMA also included 14 samples of nontumor brain (NB). Eight additional GBM samples from the ACCCC were included for the transcriptome analysis (Table S1). For western blots, three grade II, two grade III, and four grade IV fresh glioma samples from ACCCC were included (Table S1). A second cohort of 49 GBMs was obtained from the Hospital do Cancer de Pernambuco (HCP) and Hospital da Restauração (HR), Recife, Brazil (Table S1). The experiments were undertaken with the understanding and written consent of each subject. The study methodologies were approved by Ethical Committees of the ACCCC (approval number 1485/10), HCP (CAAE: 5576716.3.0000.5205), and HR (CAAE: 55476716.3.3002.5198), and conformed to the standards set by the Declaration of Helsinki.

### 2.2 Immunohistochemistry

Immunohistochemistry (IHC) was performed as previously described (Alvarenga *et al.*, 2013). Briefly, sections were deparaffinized and hydrated and epitope retrieval was performed in a pressure cooker. Nonspecific staining was blocked by the use of Dako Protein Block (Dako, Agilent, Santa Clara, CA, USA). Sections were incubated with anti‐RSK1 (sc‐231; Santa Cruz Biotechnology, Dallas, TX, USA) at 1 : 200 dilution, anti‐RSK2 (#9340; Cell Signaling, Danvers, MA, USA) at 1 : 50 dilution, anti‐RSK3 (sc‐1431; Santa Cruz) at 1 : 100 dilution, anti‐P(S380)‐RSK (#9341; Cell Signaling) at 1 : 25 dilution, anti‐CD68 (sc‐70761; Santa Cruz Biotechnology) at 1 : 100 dilution, anti‐ lysosomal protein transmembrane 5 (LAPTM5; ab108017; Abcam, Cambridge, UK) at 1 : 50 dilution, and anti‐IDH1^R132H^ (DIA‐H09; Dianova, Hamburg, Germany) at 1 : 100 dilution, in 1% BSA in PBS for 18 h at 4 °C in a humidity chamber. Secondary antibody staining was performed using EnVision + Dual Link (Dako). Color was developed by DAB. As positive controls, we included tissues with known protein expression and the primary antibody was omitted for negative controls. Quantification of HSCORE was digitally made by Aperio ScanScope XT (Leica, Buffalo Grove, IL, USA) as previously described (Alvarenga *et al.*, 2013). Briefly, each pixel was classified as negative (0), weakly positive (1), positive (2), or strongly positive (3). The number of pixels in each category was then counted and a HSCORE was calculated according to the formula HSCORE = Σ(*i* × Pi), where Pi = percentage of positive pixels, varied from 0% to 100%, and pixel staining intensity *i* = 0, 1, 2, or 3 (Hatanaka *et al.*, 2003). For IDH1^R132H^ IHC, samples with no staining were considered wild‐type, and when staining of any intensity was observed, the samples were considered IDH1 mutant (Machado *et al.*, 2018). For CD68 staining, the % of strongly labeled pixels was measured.

### 2.3 Western blot

Fresh glioma samples were processed after surgery without a freezing step. The GBM cell line LN‐18 (ATCC^®^ CRL‐2610, Manassas, VA, USA) was used as a control. LN‐18 cells were serum‐starved for 48 h followed by 10% serum treatment for 15 min. LN‐18 cells were also transfected to express HA‐RSK3 or HA‐RSK4 as in Roffé *et al. *(2015). Tissue and cell extracts were obtained and used for western blot as in Roffé *et al. *(2015). In the case of tissue samples, the lysates were performed with the help of a polytron. The antibodies for RSK1, RSK2, RSK3, and P(S380)‐RSK were the same of the IHC. Additional antibodies were as follows: RSK4 (sc‐100424; Santa Cruz), P(S227)‐RSK2 (#9341; Cell Signaling), PTEN (#9559; Cell Signaling), and ERK1/2 (#9102; Cell Signaling).

### 2.4 Immunohistofluorescence

For multiplex immunohistofluorescence, the Opal™ 4‐Color Manual Kit (PerkinElmer, Waltham, MA, USA) was used according to the manufacturer's instructions. Briefly, slides were deparaffinized and rehydrated and epitope retrieval was performed by microwave in AR6 buffer. Endogenous peroxidase activity and nonspecific binding were blocked. Incubation with primary antibodies (CD68; Santa Cruz, sc‐70763, 1 : 5000; LAPTM5; Abcam, ab108014, 1 : 100; RSK1 Santa Cruz, sc‐231, 1 : 5000), diluted in Tris/HCl (50 mm) pH 7.5 + 1% BSA, was performed overnight at 4 °C. After washing, secondary antibody Advance HRP Link (Dako^®^‐K4068) was used. The fluorophore of the Opal working solution diluted 1 : 1000 in 1X amplification diluent was applied, and the slides were incubated for 10 min. Removal of the antibodies was performed with AR6 buffer in microwave, followed by a second round of primary and secondary antibodies. Nuclear staining was performed with Draq5 (Thermo Fisher, Waltham, MA, USA). Images were acquired by Leica SP5 confocal microscopy (Leica).

### 2.5 HTA 2.0 microarray processing and analysis

mRNA from the 30 GBM samples from the ACCCC cohort was extracted from FFPE slices using the ReliaPrep™ FFPE Total RNA Miniprep System (Promega, Madison, WI, USA) and processed using the SensationPlus™ FFPE Amplification and 3′ IVT Labeling Kit (Thermo), followed by GeneChip Human Transcriptome Array 2.0 (Thermo) according to the manufacturer’s instructions. The CEL files were normalized and summarized at the gene level using SST‐RMA algorithm from the transcriptome analysis console software (Thermo). Normalized expression and DABG (detection above background) values were retrieved from the CHP files using the affxparser package. Five of the 30 FFPE blocks were newer, and a batch effect was observed for them. Thus, ComBat algorithm was used to remove batch effects (Leek *et al.*, 2012). Transcript clusters with DABG ≥ 0.05 for all of the samples were excluded. Data were annotated according to the hta20transcriptcluster.db package in r. Only transcript clusters with a corresponding RefSeq ID for protein (‘NP’) were kept, and transcript clusters for the same gene were collapsed (based on max intensity) using the wgcna package (Langfelder and Horvath, 2008). For unsupervised hierarchical clustering, differentially expressed gene (DEG) analysis, and signature acquisition, the dataset was filtered to keep mRNAs with higher median intensity and standard deviation (sd) than the RSK1 mRNA. A total of 4562 genes were used for analysis. DEG was obtained using the limma package in r (Ritchie *et al.*, 2015). DEGs were used as input for the GOstats package to determine the biological processes enriched in RSK1^hi^ and RSK1^lo^ groups (Falcon and Gentleman, 2007). As universe for GOstats, all mRNAs from the filtered dataset were used and a hypergeometric test was applied with a pvalueCutoff = 0.001.

**Figure 1:**
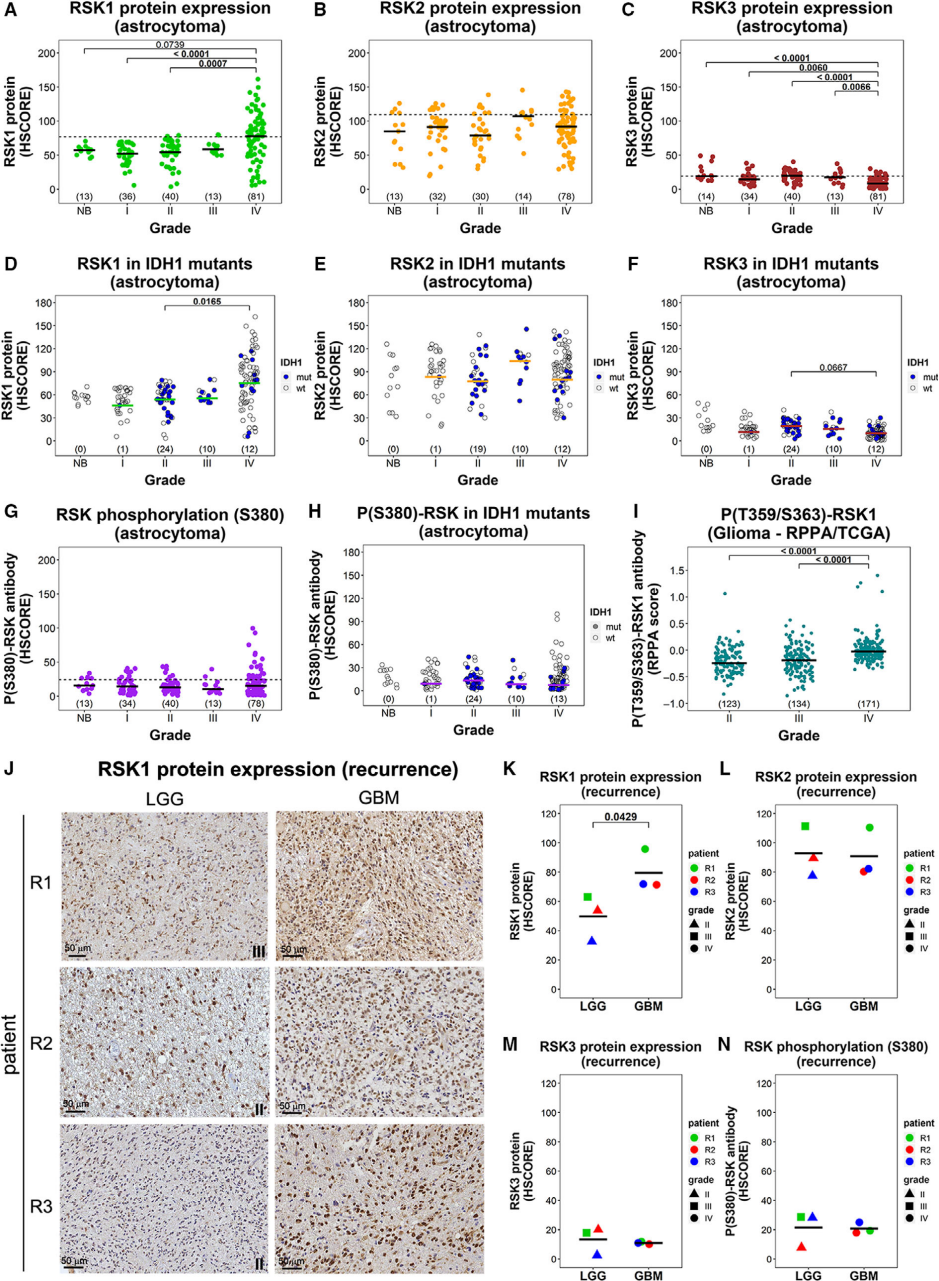
Expression of RSK isoforms in gliomas of different grades and recurrence. (A) RSK1, (B) RSK2, and (C) RSK3 protein levels and (G) P(S380)‐RSK levels in astrocytomas and nontumoral brain (NB) from the ACCCC cohort are expressed as HSCORE. The dashed line indicates the 3rd quartile for all the samples. (D) RSK1, (E) RSK2, and (F) RSK3 protein levels and (H) P(S380)‐RSK levels in samples bearing IDH1^R132H^ mutation. The wild‐type samples (empty circles) were not considered for statistics calculation. (I) Analysis of RPPA data (TCGA) for P(T359/S363)‐RSK1 antibody in LGG (grades II and III) and GBM (grade IV). P(T359/S363)‐RSK1 levels were compared for grade II, grade III, and grade IV (GBM). The number of samples in each grade is indicated in parentheses. (J) IHC reactions for RSK1 in matched samples of LGG and the corresponding GBM recurrence from three different patients. The grade for the corresponding LGG is indicated. Scale bars: 50 μm. (K–N) Quantification of (K) RSK1, (L) RSK2, and (M) RSK3 protein levels and (N) P(S380)‐RSK levels for the cases shown in (J). The *P*‐value for a paired sample *t*‐test is indicated.

### 2.6 Profiling of immune infiltrate based on transcriptome data

We used the estimate package to estimate the nontumoral fraction, and immune and stromal components for the 30 GBM samples (Yoshihara *et al.*, 2013). The profiling of 22 immune‐related cell types was performed using the LM22 gene signature for the CIBERSORT software (https://cibersort.stanford.edu/) (Newman *et al.*, 2015). For CIBERSORT, the normalized transcriptome data were transformed by applying 2^data. We disabled quantile normalization, and 1000 permutations were carried on. The relative formula of immune cells was corrected with the tumor purity estimation from the estimate analysis, to allow determination of the fraction of tumor represented by each cell type.

### 2.7 Determination of signature enrichment scores by GSVA

The Gene set variation analysis package for r was used with the following parameters: mx.diff = T and method = ‘gsva’ (Hänzelmann *et al.*, 2013). Before using GSVA, mRNAs with a median intensity and sd below any mRNA from the signature were excluded. The GBM molecular subtype was determined using the gene sets provided in the GSEA webpage (http://software.broadinstitute.org/gsea) corresponding to the TCGA 2010 signatures (Verhaak *et al.*, 2010). A newer (2016) set of signatures was reported by TCGA for IDH wild‐type GBMs, where the neural subtype was eliminated (Ceccarelli *et al.*, 2016).

### 2.8 TCGA datasets for gliomas

CEL files from GBM microarrays (GeneChip^®^ HT Human Genome U133 Array Plate Set; Affymetrix, Santa Clara, CA, USA) were obtained from TCGA portal (https://portal.gdc.cancer.gov/) and processed using the affy (Gautier *et al.*, 2004) package in r. Arrays were normalized and summarized by robust multi‐array average (RMA), and the ComBat algorithm was used to remove batch effects (batch information was obtained from the TCGA batch effects website: https://bioinformatics.mdanderson.org/tcgambatch/). Only batch effects for batches 1 and 79 were adjusted. GBMs from the same patients were averaged. Present/absent calls were obtained by the mas5calls function, and probes classified as absent for all the samples were filtered out. The data were annotated according to the hthgu133a.db package for r. Only probes with a corresponding RefSeq ID for protein (‘NP’) were kept, and probes for the same gene were collapsed (based on max intensity) using the wgcna package. A total of 527 GBM samples were included in the analysis. For the analysis of the RSK1 signature among different grade gliomas, RNA‐seq data from the TCGA portal (https://portal.gdc.cancer.gov/) were obtained for LGG (grades II and III) and grade IV (GBM). The data were annotated according to the org.hs.eg.db package for r. Only probes with a corresponding RefSeq ID for protein (‘NP’) were kept, and reads for the same gene were collapsed (based on max intensity) using the wgcna package. We kept mRNAs with a maximum of 16 samples with 0 counts to maintain the RSK1 signature genes. The data were normalized by ‘Trimmed Mean of M‐values’ from the edger (Robinson *et al.*, 2009) package and voom transformed (limma package) before analysis. The number of samples of the dataset was as follows: grade II = 216; grade III = 237; and GBM = 137. Normalized level 4 data from the reverse‐phase protein array (RPPA) technique were downloaded from The Cancer Proteome Atlas (TCPA, https://tcpaportal.org/tcpa/index.html) for GBM. A total of 175 samples with corresponding RPPA and microarray data were used. For the analysis of P(T359/S363)‐RSK1 and RSK1/2/3 in gliomas of different grades, we used normalized level 4 data from the pan‐cancer study. The number of samples of the dataset was as follows: grade II = 123; grade III = 134; and GBM = 171.

### 2.9 Gravendeel dataset for gliomas

CEL files from microarrays (GeneChip™ Human Genome U133 Plus 2.0 Array; Affymetrix) associated with the study from Gravendeel *et al*. were obtained (http://www.ncbi.nlm.nih.gov/geo/query/acc.cgi?acc=GSE16011) and processed using the affy package in r. Only primary samples were analyzed. Arrays were normalized and summarized by RMA, and the ComBat algorithm was used to remove batch effects. Present/absent calls were obtained by the mas5calls function, and probes classified as absent for all the samples were filtered out. The data were annotated according to the hgu133plus2.db package in r. Only probes with a corresponding RefSeq ID for protein (‘NP’) were kept, and probes for the same gene were collapsed (based on max intensity) using the wgcna package. The dataset contains nontumor (NT) = 8, grade I = 8, grade II = 24, grade III = 85, and GBM = 152.

### 2.10 Overall patient survival analysis

**Figure 2:**
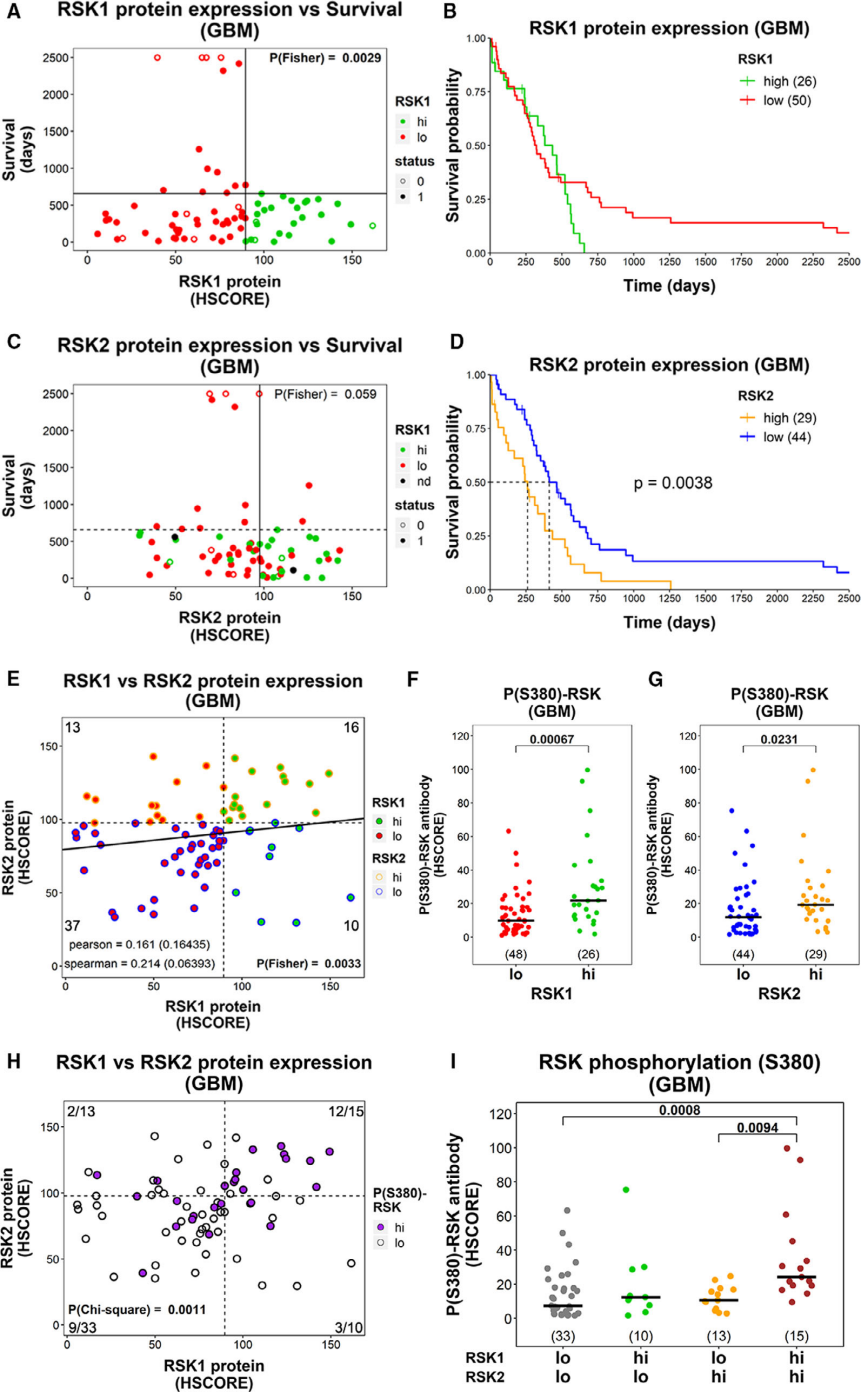
Expression of RSK isoforms in GBMs and their relationship with survival and phosphorylation. Graphs for (A) RSK1 and (C) RSK2 protein levels relative to survival in GBMs. Colors indicate whether the samples belong to RSK1^hi^ or RSK1^lo^ groups; nd = not determined. The vertical line indicates the cutoff for high‐ and low‐expression groups. The horizontal line indicates the longest survival time for RSK1^hi^. The *P*‐value of the Fisher’s exact test was calculated based on the four groups generated by the vertical and horizontal lines and is indicated. Status: 1 = dead; 0 = censored. (B,D) Overall survival plots comparing (B) RSK1^hi^ and RSK1^lo^ and (D) RSK2^hi^ and RSK2^lo^ groups. Sample number is indicated in parentheses. Proportional Hazards cannot be assumed for RSK1, and thus, the *P*‐value was not calculated in (B). (E) Correlation between RSK1 and RSK2 protein expression in GBMs. The cutoff for high‐ and low‐expression groups is indicated by a vertical line for RSK1 and a horizontal line for RSK2. The number of samples in each quadrant and the *P*‐value for the Fisher’s exact test are indicated. (F,G) P(S380)‐RSK levels for (F) RSK1^hi^ and RSK1^lo^ groups, and (G) RSK2^hi^ and RSK2^lo^ groups. The *P*‐value for the Mann–Whitney test is indicated. The number of samples in each group is indicated in parentheses. (H) Samples were classified in P(S380)‐RSK^hi^ (purple) and P(S380)‐RSK^lo^ (white) and indicated in a graph of RSK1 vs. RSK2 protein expression. The cutoff for the separation in high‐ and low‐expression groups is indicated by a vertical line for RSK1 and a horizontal line for RSK2. The number of P(S380)‐RSK^hi^ samples/total number of samples is indicated in each quadrant. The *P*‐value for chi‐square test is indicated. (I) P(S380)‐RSK levels for RSK1^lo^‐RSK2^lo^, RSK1^hi^‐RSK2^lo^, RSK1^lo^‐RSK2^hi^, and RSK1^hi^‐RSK2^hi^ GBMs. The number of samples in each group is indicated in parentheses.

**Figure 3:**
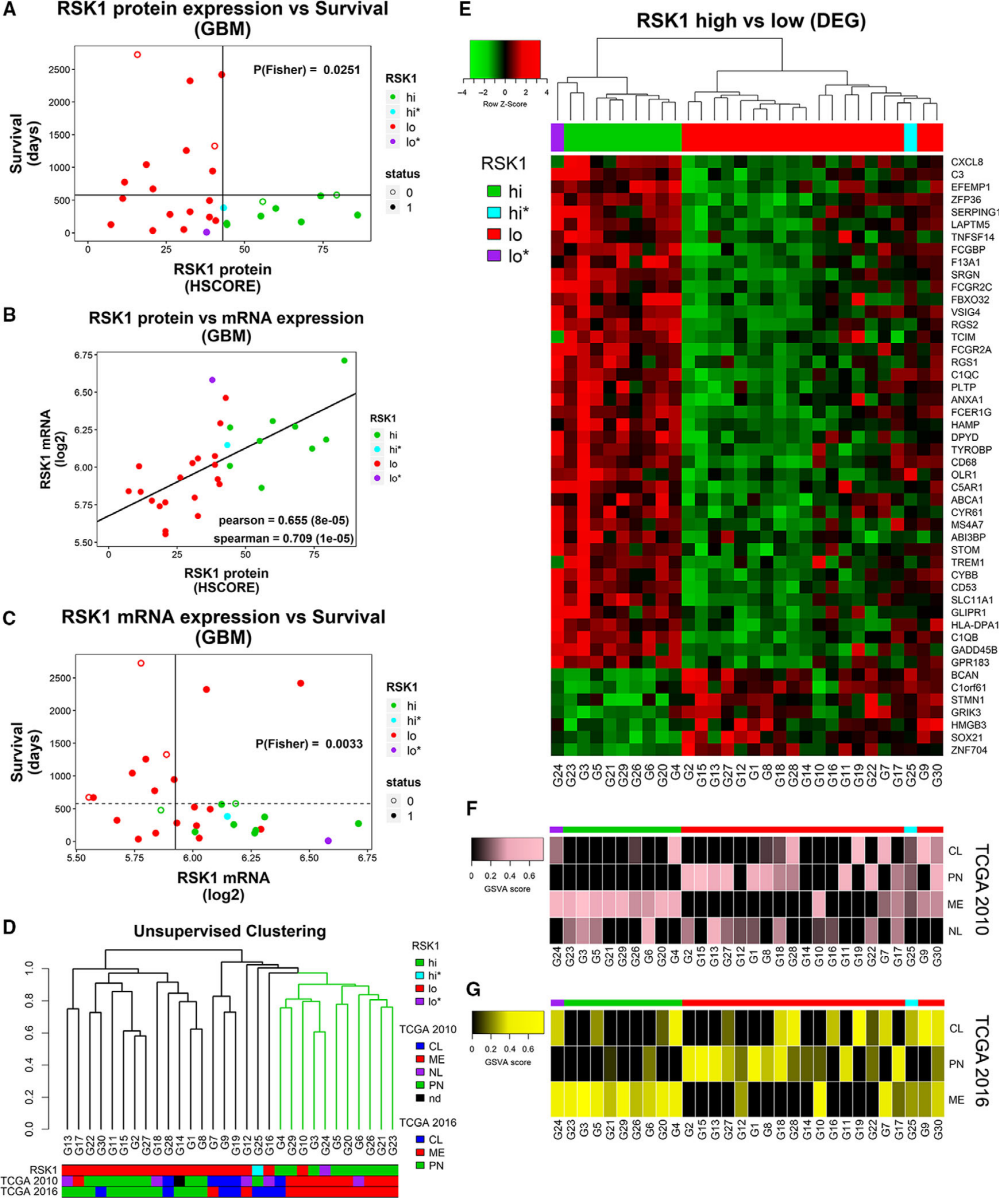
RSK1‐associated transcriptome in GBMs. (A) RSK1 protein levels (HSCORE) for 30 GBM samples (ACCCC) relative to patient survival. The horizontal line indicates the longest survival time for RSK1^hi^ group. The *P*‐value of the Fisher’s exact test was calculated based on the four groups generated by the vertical and horizontal lines and is indicated. Status: 1 = dead; 0 = censored. (B) Comparison of protein and mRNA (microarray) RSK1 levels. (C) Relationship among RSK1 mRNA levels and survival. Colors indicate whether the samples belong to RSK1^hi^ or RSK1^lo^ groups. RSK1^hi*^ and RSK1^lo*^ samples showed ambiguity between RSK1 protein levels and transcription program. The vertical line indicates the cutoff for high‐ and low‐expression groups, and the horizontal line indicates the longest survival time for RSK1^hi^ group. The *P*‐value of the Fisher’s exact test was calculated based on the four groups generated by the vertical and horizontal lines and is indicated. Status: 1 = dead; 0 = censored. (D) Unsupervised hierarchical clustering based on the transcriptome of the GBM samples. (E) Heatmap showing the DEGs between RSK1^hi^ and RSK1^lo^ groups (FDR < 0.015 and |logFC| ≥ 0.89). Clustering was performed using the DEGs. (F, G) The GSVA score indicating enrichment for each subtype is shown in (F) for TCGA 2010 and (G) TCGA 2016 signatures. CL, classical; ME, mesenchymal; NL, neural; nd: not determined; PN, proneural.

Survival for ACCCC cohort was calculated using the date of the surgery as initial time, due to the lack of the date of image diagnostic for some of the samples. For the Recife cohort, consisting of recent cases, the image diagnosis date was available and used as initial time for survival calculation. The analysis and survival curves were performed using survminer and survival packages in r. We used the overall survival data for the definition of high‐ and low‐expression groups (or enriched and underrepresented RSK1 signature groups). For RSK1 in the ACCCC, its expression levels (HSCORE) were analyzed regarding survival. Two cutoffs that minimized the *P*‐value of a Fisher’s exact test were set: one dividing the samples in RSK1^hi^ and RSK1^lo^, and the other in short and long survivors (Fig. 2A, Fig 3A). The latter corresponded to the longest survival time of the RSK1^hi^ group. For the remaining markers, including RSK1 in the Recife cohort, we determined the log‐rank test *P*‐values for all the sets of two groups that can be formed according to expression (or signature enrichment) levels, where all the expression levels of the low‐expression group are smaller than those of the high‐expression group. Then, the distribution of samples that allowed lowest *P*‐values while equilibrating the number of members in each group and maximizing the median‐average survival differences was chosen. The separation of groups is indicated with a vertical line in the graphs of expression (or RSK1 signature enrichment) vs. survival. To calculate the Fisher’s exact test for markers of the ACCCC and Recife cohorts, the survival time cutoff was set to the longest survival time of the RSK1^hi^ group. For the TCGA and Gravendeel cohorts, after the determination of the signature enrichment cutoff, the survival time limit was set as the one minimizing the *P*‐value of a Fisher’s exact test. The censored samples below the survival time cutoff were not considered for the Fisher’s exact tests.

### 2.11 Statistical analysis

Kruskal–Wallis test followed by Dunn’s multiple comparison test was applied using the dunn.test package in r. Univariate survival analyses were performed using the Kaplan–Meier estimator and the log‐rank test (*P*‐values are indicated in the plots), and median survival is shown in Table S2. Multivariate Cox regression analysis was performed with the survival package for r. In cases where proportional Hazards could not be assumed for a covariate (tested by the cox.zph function), univariate analysis was performed after stratification by that same covariate. Fisher’s exact test was used for significance testing in experiments with a 2 × 2 contingency table (*P*‐values are indicated in the graphs). Pearson’s and Spearman’s coefficients for correlations are indicated in the graphs along with the *P*‐values in parentheses. Values of *P* < 0.05 were considered significant. All statistical analysis was performed in rstudio with r version 3.5.1.

### 2.12 Graphics

Graphs were assembled using the ggplot2 package in rstudio with r version 3.5.1. For unsupervised hierarchical clustering, we used Pearson’s distance and average linkage. The dendrogram was optimized with the dendextend package (Galili, 2015). The heatmap.2 function in r was used to generate the DEG heatmap. Default options were used for clustering. Appendix S1 includes additional information of r packages used in this article.

## 3 Results

### 3.1 RSK1, but not RSK2 or RSK3, protein levels are higher in grade IV gliomas

RSK1 protein levels were evaluated by IHC in a TMA containing NB and grade I‐IV astrocytomas from the ACCCC. While the median RSK1 protein expression was higher in GBM (grade IV) cases than the median of NB and LGG (grades I–III) cases (Fig. 1A, Fig. S1A), only about half of GBMs showed higher RSK1 levels than NB and LGG. The other half of GBMs expressed RSK1 at levels comparable to the rest of the gliomas. A different subset of gliomas was tested for RSK1 protein by western blot showing an expression pattern compatible with the result of the TMA (Fig. S2A). On the other hand, RSK2 protein expression did not differ significantly among NB and the various grades of gliomas, showing overlapping distributions of intensities (Fig. 1B, Fig. S1B). While some samples present very low or undetectable levels of RSK1, RSK2 can be easily detected in low‐expressing samples even by western blot (Fig. S2B). RSK3 isoform levels were very low when compared to RSK1 and RSK2, and GBMs show lower RSK3 levels than NB and LGG (Fig. 1C, Fig. S1C). Detection of RSK3 in gliomas by western blot required very long exposure times (Fig. S2C) and is consistent with the apparent lack of RSK3 expression in GBM cell lines (Roffé *et al.*, 2015). We were not able to detect RSK4 in both gliomas (Fig. S2C) and GBM cell lines (Roffé *et al.*, 2015) by western blot. The IDH1 mutation occurs frequently in grade II and III gliomas, and indeed, when considering only the samples positive for IDH1^R132H^ (Fig. S1E), the median RSK1 levels were also higher in GBMs than in LGG (Fig. 1D). Also in IDH1 mutant samples, the levels of RSK2 and RSK3 were comparable between the grades (Fig. 1E,F). This strongly suggests that increase in the RSK1 isoform can also occur during progression of secondary GBMs.

We included in our analysis an antibody originally raised to detect RSK1 when it is phosphorylated at S380 (Fig. S1D, Fig. S2B). It can also detect RSK2 and RSK3 when phosphorylated at a homologous serine (according to the vendor; Cell Signaling). This phosphorylation is necessary for RSK activation (Anjum and Blenis, 2008). Median levels of RSK phosphorylation (S380) did not differ significantly among NB and the various grades of gliomas (Fig. 1G,H). However, high levels of P(S380)‐RSK were detected in a subgroup of GBMs, consistent with the high levels of RSK1 observed. In addition, we evaluated P(T359/S363)‐RSK1 levels, also required for activation (Anjum and Blenis, 2008), in grade II‐IV gliomas using the RPPA data from TCGA (Brennan *et al.*, 2013). According to Cell Signaling, the P(T359/S363)‐RSK1 antibody (#9344) does not cross‐react with RSK2 but can show some reaction with RSK3. Our analysis revealed increased levels of P(T359/S363)‐RSK1 in GBMs (Fig. 1I). Since RSK3 is essentially absent, we can confirm that RSK1 activation is augmented in GBMs. In conclusion, increased RSK1 expression and activation is a feature that appears in a subgroup of GBMs.

The ACCCC cohort included three cases of recurrence with matched LGG and GBM samples. RSK1 expression levels increased after the recurrence in all the cases (Fig. 1J,K); however, RSK2, RSK3, and P(S380)‐RSK levels did not (Fig. 1L–N). Altogether, these observations suggest that an isoform‐specific RSK1 increase can occur during the progression of gliomas from low to high grade.

### 3.2 High RSK1 and RSK2 protein levels in GBM are associated with worse survival

RSK1 and RSK2 levels were analyzed relative to survival outcomes in GBMs. We found that the 26 (34.2%) GBMs expressing the highest levels of RSK1 did not survive more than 1.8 years (horizontal line in Fig. 2A,B), and named this group RSK1^hi^. The remaining 2/3 of GBMs (named RSK1^lo^ group) included 14/50 cases with survival times longer than 1.8 years, seven of them being very long survivors (> 3 years). IDH1^R132H^ GBMs were present in the RSK1^hi^ group, suggesting that secondary GBMs can also express high RSK1 levels (Fig. S3A). The 29 GBMs expressing the highest RSK2 levels showed significantly worse survival than the remaining 44 (vertical line separates RSK2 groups in Fig. 2C,D). In contrast to RSK1^hi^ group (Fig. 2A), two of the 11 GBMs expressing the highest RSK2 levels showed survival > 1.8 years. Although not significant, we observed that while 10/40 (25%) of RSK2^lo^ GBMs were IDH1^R132H^, only 2/29 (6.9%) of RSK2^hi^ were IDH1^R132H^ (P_Fisher_ = 0.0604; Fig. S3B). There was no correlation between RSK1 and RSK2 expression in GBMs, and while 16/29 (55.2%) of RSK2^hi^ GBMs belong to RSK1^hi^ group, 16/26 (61.5%) of RSK1^hi^ GBMs belong to RSK2^hi^ group (Fig. 2E). We could not apply a multivariate Cox analysis for RSK2 and RSK1 groups as covariates because it was not possible to assume proportional Hazards for RSK1 (*P* = 0.0101 for cox.zph function). However, RSK2 remained a significant predictor after stratification by RSK1 groups (Fig. S4A). Multivariate cox analysis indicated that RSK2 was an independent prognostic factor when adjusted for IDH1^R132H^ status (Fig. S4B) or chemotherapy (CTx), when the latter analysis was performed stratifying by radiotherapy (RTx; Fig. S4C). In conclusion, for this cohort, high levels of RSK1 were associated with the absence of long survivors and RSK2 was an independent predictor of poor survival.

P(S380)‐RSK levels were higher in RSK1^hi^ group (median = 21.74) than in RSK1^lo^ (median = 9.81, *P* = 0.00067; Fig. 2F). Although less significant, P(S380)‐RSK levels were also higher in RSK2^hi^ group (median = 19.15) than in RSK2^lo^ (median = 11.84, *P* = 0.0231; Fig. 2G). For P(S380)‐RSK, we defined a group of 28/74 GBMs showing the highest phosphorylation levels while resulting in the lowest *P*‐value for the Fisher’s exact test when considering 1.8 years as the limit for long and short survivors (vertical and horizontal lines in Figs S5A,B and S3C). 16/26 (61.5%) of RSK1^hi^ GBMs were also P(S380)‐RSK^hi^, but only 10/47 (21.2%) of P(S380)‐RSK^lo^ GBMs were RSK1^hi^ (Fig. S5C). On the other hand, 15/29 (51.7%) of RSK2^hi^ GBMs were also P(S380)‐RSK^hi^ and 14/45 (31.1%) of P(S380)‐RSK^lo^ GBMs were RSK2^hi^ (Fig. S5D). Interestingly, 12/15 (80%) GBMs belonging to both RSK1^hi^ and RSK2^hi^ groups at the same time (RSK1^hi^‐RSK2^hi^) were P(S380)‐RSK^hi^, in contrast to the representation of P(S380)‐RSK^hi^ GBMs in RSK1^hi^‐RSK2^lo^ (3/10 or 30%), RSK1^lo^‐RSK2^hi^ (2/13 or 15.4%), and RSK1^lo^‐RSK2^lo^ (8/32 or 25%; Fig. 2H) subgroups. In fact, the P(S380)‐RSK levels were significantly higher for RSK1^hi^‐RSK2^hi^ GBMs than for RSK1^lo^‐RSK2^lo^ and RSK1^lo^‐RSK2^hi^ (Fig. 2I). As mentioned above, P(T359/S363)‐RSK1 levels (RPPA experiment—TCGA) in GBMs can be considered a measure of RSK1 activation. Accordingly, higher levels of P(T359/S363)‐RSK1 were associated with a lower proportion of long survivors in GBMs from the TCGA cohort (Fig. S5E,F). In conclusion, high P(S380)‐RSK levels (i.e., RSK activation) in GBMs were associated with high levels of both RSK1 and RSK2; however, the contribution of RSK1 was more important.

### 3.3 Transcriptomic characterization of RSK1^hi^ glioblastomas

Since the RSK1^hi^ group is composed of tumors that gained a new feature (high expression of RSK1), we further characterized their transcriptome. We performed both IHC for RSK1 and microarray analysis in FFPE sections of 30 GBM cases. Twenty‐two of these samples were originally included in the TMA, and 8 were new samples (Table S1). As expected from the results of the TMA (Fig. 2A), we defined a RSK1^hi^ group consisting of 10/30 (30%) of the cases where none of the patients exceeded a survival time of 1.7 years (Fig. 3A, Fig. S6A). The mRNA levels for RSK1 obtained from the microarray correlated with protein levels (Fig. 3B). RSK1 mRNA expression allowed the separation in two groups with different overall survival; however, it did not allow for the determination of an RSK1‐mRNA^hi^ group that excluded long survivors (Fig. 3C, Fig. S6B). The transcriptome data were used to perform an unsupervised hierarchical clustering (Fig. 3D) which revealed that RSK1^hi^ GBMs clustered together, reinforcing that high levels of RSK1 are indeed associated with a distinctive characteristic. Using two different sets of molecular classification signatures from TCGA (2010 and 2016), we observed that most of the RSK1^hi^ GBMs were classified as mesenchymal, but not all the mesenchymal GBMs were RSK1^hi^ (Fig. 3D). DEGs between the RSK1^hi^ and RSK1^lo^ groups were identified using the limma package in r (Table S3) (Ritchie *et al.*, 2015). One RSK1^hi^ GBM that clustered with the RSK1^lo^ cases (RSK1^hi*^) and one RSK1^lo^ GBM that clustered with the RSK1^hi^ cases (RSK1^lo*^) were excluded from the analysis of DEGs due to their ambiguity. In Fig. 3E, DEGs are shown in a heatmap and it is evident that the RSK1^lo^ group could be further divided in two subclusters. Scores representing the enrichment of TCGA signatures for each subtype (GSVA scores) demonstrate that all of the RSK1^hi^ samples show a mesenchymal enrichment, even if the final classification differs, and the proneural signature is essentially absent (Fig. 3F,G).

### 3.4 Evidence of immune infiltration in RSK1^hi^ glioblastomas

**Figure 4:**
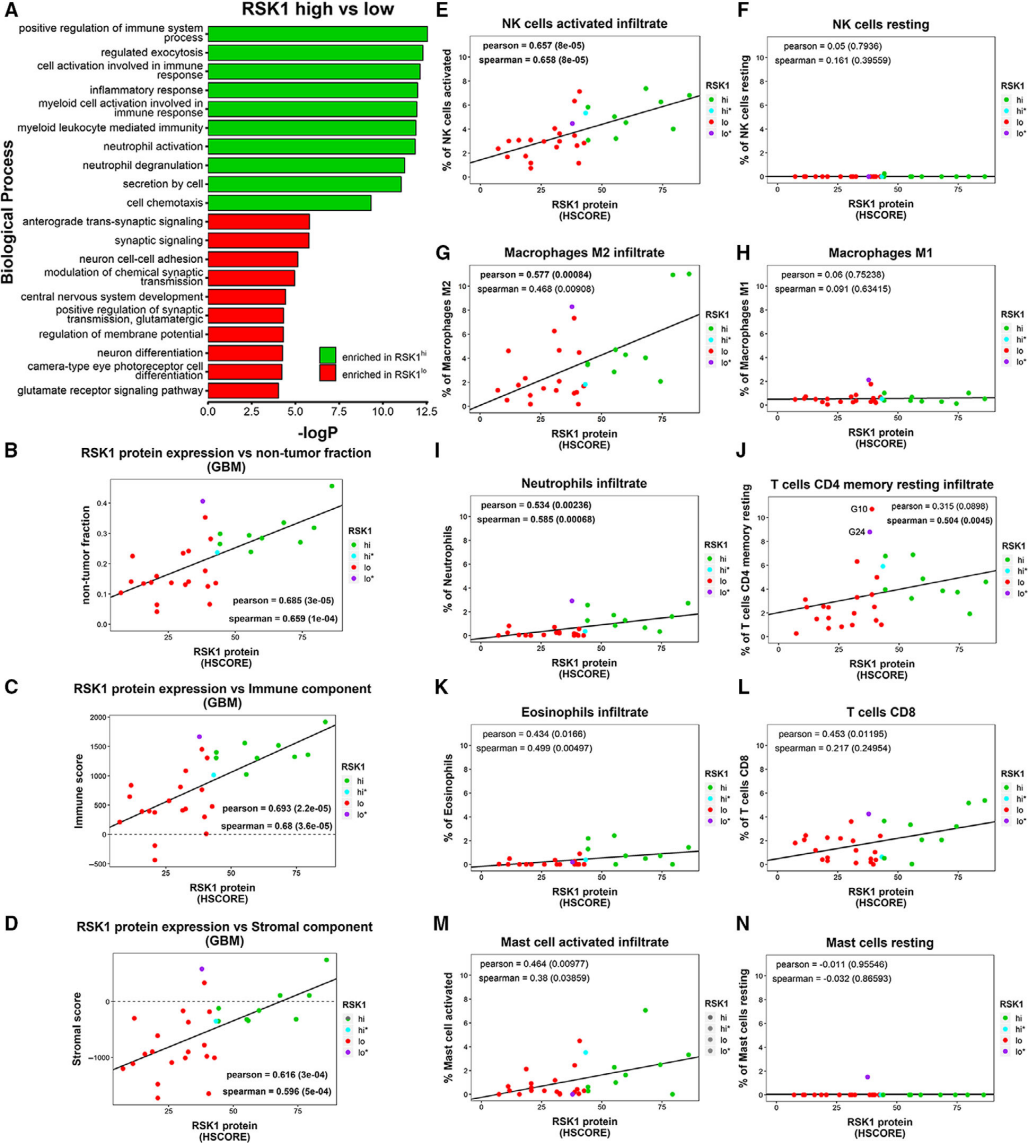
RSK1 protein expression is associated with immune infiltrate in GBMs. (A) Biological processes enriched in RSK1^hi^ and RSK1^lo^ groups, obtained with the gostats package For this graph, biological processes with sizes of more than 500 or less than five genes were excluded. (B–D) Relationship between RSK1 protein expression (HSCORE) and (B) nontumor fraction, (C) immune component, and (D) stromal component of GBMs. The estimate package was used. (E–N) The fraction of immune‐related cells for each GBM was estimated by CIBERSORT using the LM22 signature. The immune cell types and their correlation with RSK1 protein expression are shown. Samples G10 and G24 are labeled in J.

We evaluated the biological processes associated with the RSK1^hi^ and RSK1^lo^ groups using the DEGs as input for the gostats package (Table S4) (Falcon and Gentleman, 2007). While the RSK1^hi^ group showed enrichment in immune‐associated processes, RSK1^lo^ GBMs showed enrichment in processes of the nervous system (Fig. 4A). We next applied the estimate algorithm to estimate the nontumoral fraction of GBMs based on transcriptome data. Strikingly, RSK1 protein levels strongly correlated with the presence of nontumor components (Fig. 4B), being mainly composed of immune infiltrate (Fig. 4C). Of note, only 5/30 GBMs showed a positive score for stromal component (Fig. 4D). We performed a CIBERSORT analysis using the gene signature of 22 immune cell types (LM22) (Newman *et al.*, 2015). The relative immune cell composition was corrected by the fraction of nontumor component from the estimate package to calculate the contribution of each cell type to the tumor (Fig. 4E–N, Fig. S7). RSK1 protein expression was strongly correlated with the percent of activated natural killer (NK) cells (Fig. 4E). Other cell types that were found to correlate with RSK1 levels were macrophage M2 (Fig. 4G), neutrophils (Fig. 4I), resting‐memory CD4 T cells (Fig. 4J), eosinophils (Fig. 4K), CD8 T cells (Fig. 4L), and activated mast cells (Fig. 4M). Two samples, G24 (RSK1^lo*^) and G10 (Fig. 3D), showed an exaggerated enrichment in resting‐memory CD4 T cells pointing to mesenchymal GBMs with a different type of infiltrate (Fig. 4J). Essentially, no immune cell type was enriched in the RSK1^lo^ group.

### 3.5 RSK1 protein levels associate with LAPTM5 expression and CD68^+^ infiltrate

**Figure 5:**
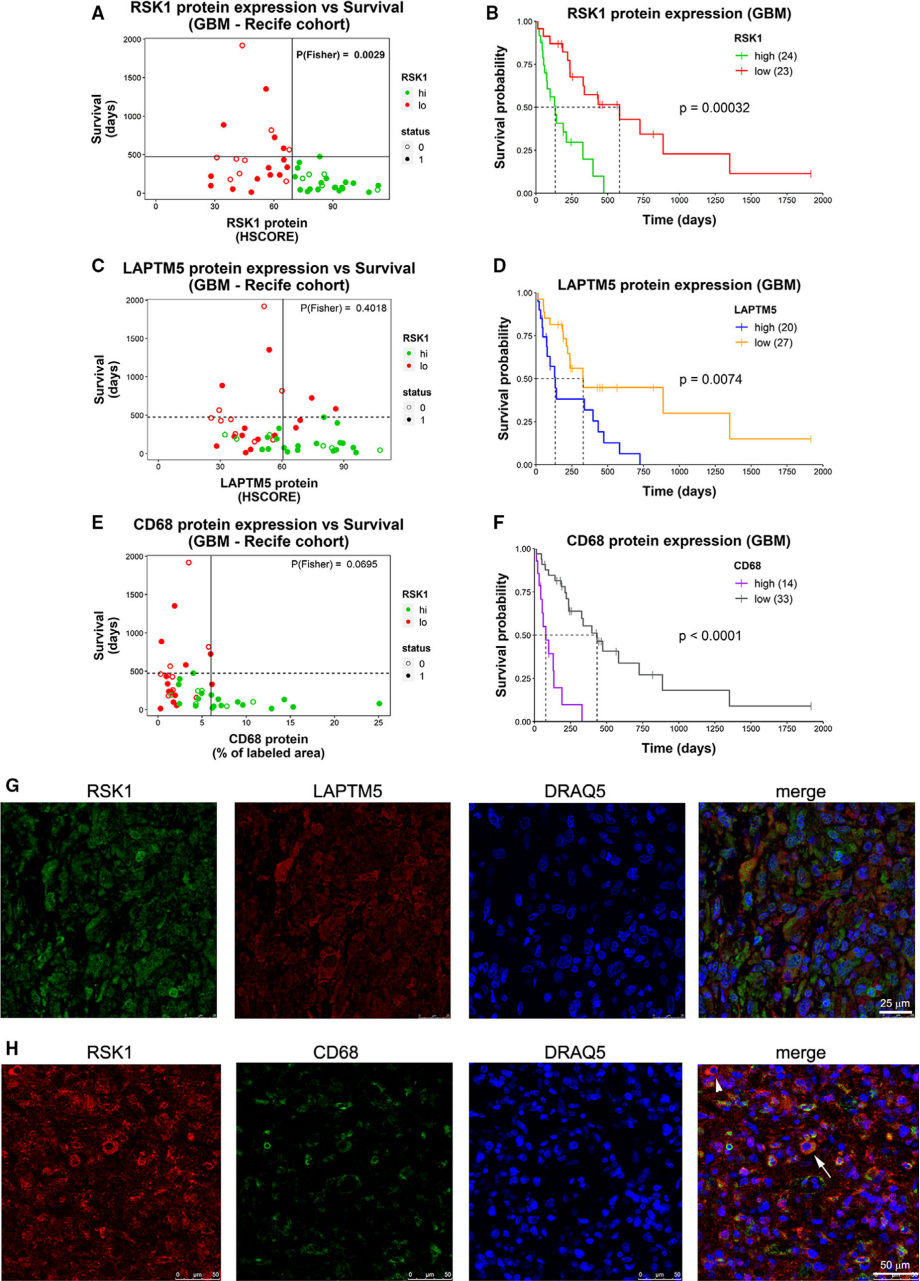
RSK1 protein expression is associated with LAPTM5 and CD68 expression in GBMs. Protein levels for (A) RSK1 (HSCORE), (C) LAPTM5 (HSCORE), and (E) CD68 (% of labeled area) from the Recife cohort are shown relative to patient survival. Colors indicate whether the samples belong to RSK1^hi^ or RSK1^lo^ groups. The vertical line indicates the cutoff for high‐ and low‐expression groups, and the horizontal line indicates the longest survival time for RSK1^hi^ group. The *P*‐value for the Fisher’s exact test was calculated based on the four groups generated by the vertical and horizontal lines and is indicated. Status: 1 = dead; 0 = censored. (B, D, F) Overall survival plots comparing (B) RSK1^hi^ and RSK1^lo^, (D) LAPTM5^hi^ and LAPTM5^lo^, and (F) CD68^hi^ and CD68^lo^ groups. The number of samples is indicated in parentheses. (G) Multiplex immunohistofluorescence detection of RSK1, LAPTM5, and nuclei labeling (DRAQ5) in RSK1^hi^ GBM tissue. Scale bars: 25 μm. (H) Multiplex immunohistofluorescence detection of RSK1, CD68, and nuclei labeling (DRAQ5) in RSK^hi^ GBM tissue. Arrowhead points to a CD68^‐^ RSK1^+^ cell. Arrow points to a CD68^+^ RSK1^+^ cell. Scale bars: 50 μm.

An independent cohort of GBMs was included to confirm our observations (Recife cohort, Table S1). IHC for RSK1 (Fig. S8A) resulted in the same distribution regarding survival that we observed in the ACCCC cohort (compare Fig. 5A with Fig. 2A), suggesting the existence of three populations: (a) high RSK1 levels/short survival; (b) low RSK1 levels/short survival; and (c) low RSK1 levels/long survival. This casuistry showed a reduced proportion of short survivor cases in the RSK1^lo^ group when compared to the ACCCC cohort, and we could assume proportional Hazards. Thus, we defined an RSK1^hi^ group (24/47 cases) that excluded long survivors and showed worse survival than the RSK1^lo^ group (23/47 cases, Fig. 5B). The mRNA for LAPTM5 belongs to TCGA 2010 mesenchymal signature (Verhaak *et al.*, 2010), and its function was related to the immune system (Glowacka *et al.*, 2012; Ouchida *et al.*, 2010; Ouchida *et al.*, 2008). Since most RSK1^hi^ GBMs show mesenchymal signature enrichment, we decided to analyze LAPTM5 protein levels by IHC (Fig. S8B). We defined two groups according to LAPTM5 expression, where LAPTM5^hi^ GBMs showed worse survival; however, not all the long survivors were excluded from this group (Fig. 5C,D). Although RSK1 and LAPTM5 levels were highly correlated, we found a fraction (8/25; 32%) of RSK1^hi^ GBMs being LAPTM5^lo^ (Fig. S8D), reinforcing our suggestion that not all RSK1^hi^ GBMs are mesenchymal. CD68 is considered a macrophage marker that in gliomas is mainly derived from M2 macrophages (Prosniak *et al.*, 2013). However, CD68 can be expressed in other cell types (Gottfried *et al.*, 2008). We defined two groups according to CD68 expression (Fig. S8C), where CD68^hi^ GBMs showed worse survival (Fig. 5E,F). CD68 was also highly correlated with RSK1 levels and to a lesser degree with LAPTM5 (Fig. S8E,F). Alike the ACCCC cohort, IDH1 mutation was present in RSK1^hi^ GBMs (Fig. S9A) as well as in the LAPTM5^hi^ and CD68^hi^ groups (Fig. S9B,C), suggesting that secondary GBMs can also express these markers. RSK1 expression levels remained an independent prognostic marker when adjusted for LAPTM5 or CD68 in a multivariate Cox analysis (Fig. S9D,E). RSK1 was also independent of RTx and CTx, which are known prognostic covariates for GBMs (Fig. S9F). One important question is whether immune infiltrate cells can contribute to RSK1 gain in GBMs. Double labeling for RSK1 and LAPTM5 in GBM tissues revealed widespread RSK1 labeling where most RSK1^+^ cells were also LAPTM5^+^ (Fig. 5G). In the case of CD68, RSK1^+^ cells included both CD68^+^ and CD68^‐^ (Fig. 5H, Fig. S10). This suggests that RSK1 gain can originate from both immune and GBM cells.

### 3.6 Validation of a RSK1 signature to explore datasets of glioblastomas

**Figure 6:**
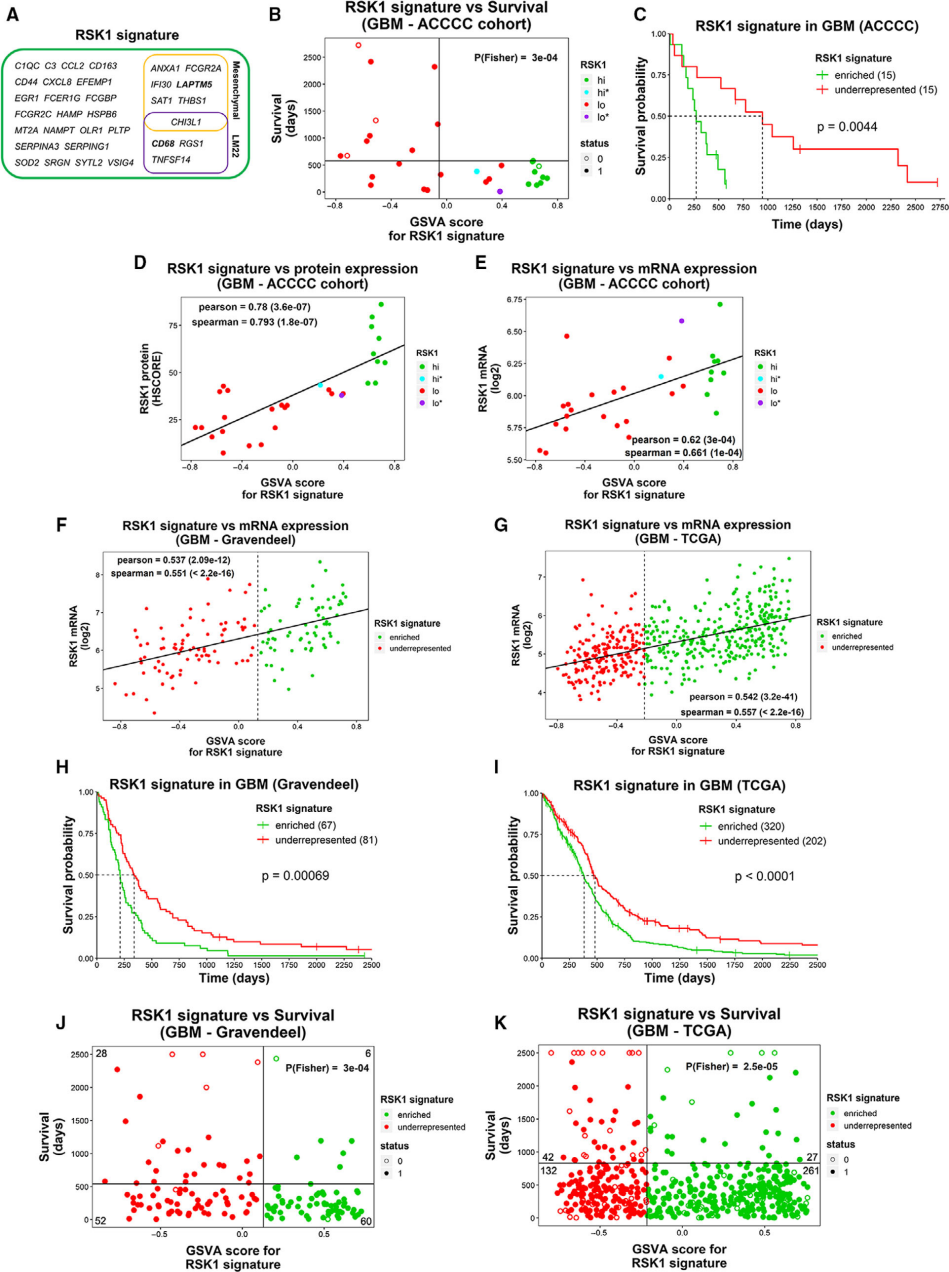
RSK1 signature can infer RSK1 levels from GBM transcriptome data. (A) The 33 genes of the RSK1 signature. Genes for which the RSK1 signature is shared by the mesenchymal subtype signature from TCGA (2010 and 2016) and/or LM22 signatures are indicated. (B) Relationship between GSVA scores for the RSK1 signature and patient survival. Colors indicate whether the samples belong to RSK1^hi^ or RSK1^lo^ groups defined in Fig. 2A. The vertical line indicates the separation of GBMs with GSVA score > −0.05 (signRSK1^enriched^) and < −0.05 (signRSK1^underrepresented^), and the horizontal line indicates the longest survival time for RSK1^hi^ group. The *P*‐value of the Fisher’s exact test was calculated based on the four groups generated by the vertical and horizontal lines and is indicated. (C) Overall survival plot comparing signRSK1^enriched^ and signRSK1^underrepresented^ groups. The number of samples is indicated in parentheses. (D) RSK1 protein (HSCORE) and (E) RSK1 mRNA (microarray) levels correlate with GSVA scores for RSK1 signature in the 30 GBM originally used to obtain the signature (ACCCC). (F, G) The RSK1 signature correlates with RSK1 mRNA levels from two public cohorts: (F) Gravendeel and (G) TCGA. The dashed line indicates the GSVA score cutoff for the survival curve in H and I. (H,I) Overall survival plots for (H) Gravendeel and (I) TCGA. The number of samples is indicated in parentheses. (J,K) Relationship between GSVA scores for the RSK1 signature and patient survival in (J) Gravendeel and (K) TCGA cohorts. The vertical line indicates the cutoff for signRSK1^enriched^ and signRSK1^underrepresented^ groups, and the horizontal line indicates the cutoff for long and short survival times. The *P*‐value of the Fisher’s exact test was calculated based on the four groups generated by the vertical and horizontal lines and is indicated along with the number of samples in each quadrant (censored samples below the survival time cutoff were not included). Status: 1 = dead; 0 = censored.

A second round of limma analysis with the previously determined DEGs for RSK1 groups (Fig. 3E) and including RSK1^hi*^ and RSK1^lo*^ samples was run to select a subpopulation of DEGs that better relates with the RSK1 protein levels. We evaluated the enrichment of subsets of upregulated mRNAs (logFC > 1.12 , FDR < 0.022) in the 30 GBM samples and obtained a signature that allowed the separation between RSK1^hi^ GBMs and long survivors (belonging to RSK1^lo^ group) using transcriptomic data (Fig. 6A,B). This RSK1 signature allowed for the reclassification of samples from the RSK1^lo^ group with RSK1 protein expression levels close to the cutoff (Fig. 6B). Accordingly, the survival of GBMs with higher GSVA scores, which indicates the signature enrichment, was significantly worse than GBMs with low GSVA score (compare Fig. 6C with Fig. S6A). The GSVA score was linearly related to RSK1 protein levels obtained by IHC (Fig. 6D) and, as expected, correlated to a lesser degree with RSK1 mRNA levels (Fig. 6E). From the 547 genes of the LM22 signature, 4 were present in the RSK1 signature, including *CD68*. From the 50 genes in TCGA 2016 mesenchymal signature, only *THBS1* was present in the RSK1 signature. From the 216 genes in TCGA 2010 mesenchymal signature, six were present in the RSK1 signature, including *LAPTM5* and *THBS1*. *CHI3L1* was present in both LM22 and TCGA 2010 mesenchymal signatures (Fig. 6A).

Using the RSK1 signature, we explored two different previously published mRNA expression datasets of GBMs: TCGA (Brennan *et al.*, 2013; McLendon *et al.*, 2008; Verhaak *et al.*, 2010) and Gravendeel (Gravendeel *et al.*, 2009). The RSK1 signature (signRSK1) correlated with RSK1 mRNA levels in both datasets (Fig. 6F,G). Furthermore, the GBMs with higher GSVA scores showed worse survival (Fig. 6H,I) and long and very long survivors were underrepresented in the signRSK1^enriched^ group. In the case of the Gravendeel dataset, cases with IDH1 mutation were present in GBMs with higher GSVA scores, but in a low proportion (Fig. S11A), resembling what we observed in the ACCCC (Fig. S3A) and Recife cohorts (Fig. S9A). However, in TCGA dataset, cases with IDH1 mutation were underrepresented in GBMs with higher GSVA scores (Fig. S11B). This apparent discrepancy can be the result of the selection of primary GBMs by the TCGA making IDH1 mutation infrequent in this cohort (Brennan *et al.*, 2013). We observed that G‐CIMP GBMs (hypermethylator phenotype) were less frequent in the signRSK1^enriched^ group of GBMs in both datasets (Fig. S11C,D).

### 3.7 The RSK1 signature can determine poor survival even in low‐grade gliomas

**Figure 7:**
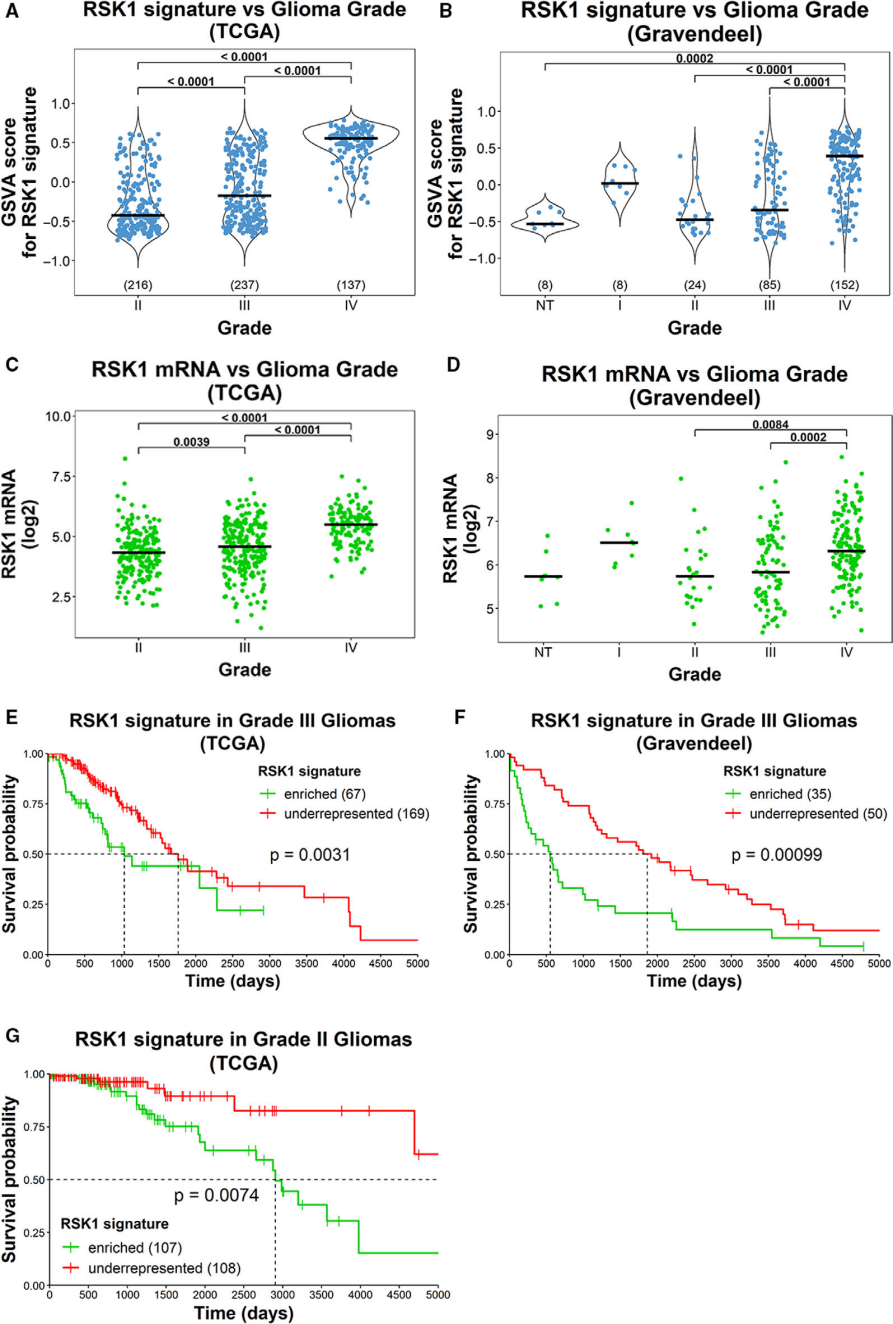
The RSK1 signature is enriched in grade IV gliomas. (A,B) GSVA scores for the enrichment in RSK1 signature (signRSK1) were obtained for gliomas of different grades from (A) TCGA and (B) Gravendeel datasets. NT = nontumor. The number of samples for each grade is indicated in parentheses. (C,D) Graphs showing the RSK1 mRNA levels for gliomas of different grades from (C) TCGA and (D) Gravendeel datasets. (E–G) Overall survival plots for (E) grade III (TCGA), (F) grade III (Gravendeel), and (G) grade II (TCGA) gliomas, comparing signRSK1^enriched^ vs. signRSK1^underrepresented^. The number of samples is indicated in parentheses.

We further validated our RSK1 signature in LGG and GBM together. RSK1 signature was enriched in GBMs when compared to LGG from TCGA (Fig. 7A). With the Gravendeel dataset, we further demonstrated that the RSK1 signature was enriched in GBMs when compared with NB (Fig. 7B). Thus, the RSK1 signature recapitulated the IHC results obtained with the RSK1 antibody (Fig. 1A). Accordingly, in both datasets we observed that RSK1 mRNA levels behaved in the same direction as the RSK1 signature (Fig. 7C,D). We also analyzed the enrichment of RSK1 signature within grade II or grade III gliomas. In both datasets, signRSK1^enriched^ grade III gliomas showed poor survival (Fig. 7E,F). The same was found for grade II gliomas from TCGA (Fig. 7G). We could not apply this analysis to the grade II gliomas from Gravendeel dataset due to an insufficient number of samples. These observations suggest that the RSK1 signature can predict RSK1‐associated events even in LGG.

## 4 Discussion

Our data demonstrate that RSK1 protein levels above those expressed in nontumoral brain define a set of GBMs with clear features. Most of the RSK1^hi^ GBMs were classified as part of the mesenchymal subtype based on TCGA signatures, which indicates that there exists an RSK‐isoform preference that accompanies a specific gene expression program. Since high levels of RSK1 are practically absent from gliomas of lower grade, RSK1 increase can be considered a hallmark of the more malignant GBMs. This proposal is further supported by the exclusion of long and very long survivors from the RSK1^hi^ group. Although IDH mutant cases can be found in the RSK1^hi^ group, it is evident that the RSK1^hi^ group is mainly composed of IDH wild‐type and non‐G‐CIMP GBMs. Furthermore, the great majority of grade II and III gliomas, where IDH mutation is prevalent, did not show high levels of RSK1.

One of the main findings of our work was the close relationship between RSK1 protein expression and immune infiltrate. It was previously observed using the same set of 22 signatures for immune cell types, LM22 (Newman *et al.*, 2015), that mesenchymal subtype GBMs showed enrichment in M2 macrophages, neutrophils, and resting‐memory CD4 T‐cell infiltrate (Wang *et al.*, 2017). Accordingly, we also observed a correlation of those cell types with RSK1 protein levels; however, the most correlated type was activated NK cells. This suggests that RSK1‐associated tumor infiltration has a partial overlap with that observed in the mesenchymal subtype. Confirming those observations, RSK1 protein levels highly correlated with the presence of CD68 in an independent GBM cohort. The presence of M2 macrophages is further indicated by the increase in RSK1^hi^ GBMs of CD163 mRNAs (Table S3), a M2 macrophage marker (Zhou *et al.*, 2015). In view of these findings, a critical question was raised on the origin of the high levels of RSK1 that appear in GBMs. Dual labeling of GBMs for RSK1 and CD68 showed that both CD68^‐^ (majority of cells) and CD68^+^ cells within the tumor express RSK1, which implies that RSK1 can indeed derive from both tumor and immune infiltrate cells. Accordingly, RSK1 expression was detected in GBM‐derived cell lines (Roffé *et al.*, 2015) and activated RSK1 can be observed in immune cells in vitro (Lin *et al.*, 2008; Zaru *et al.*, 2007). A more detailed investigation at the single cell level of GBMs, assaying different molecular markers of immune cells, will help to define the origin of RSK1. Immune system infiltration seems to play a fundamental role in the aggressiveness of the gliomas, and, accordingly, it has been proposed that a signature composed of immune‐related genes can predict the risk of gliomas (Cheng *et al.*, 2016). However, none of those genes were included in our RSK1 signature. In fact, the RSK1 signature that we used to infer RSK1 protein in datasets of GBM and LGG combines genes associated with immune infiltrate, such as *CD68*, with genes associated with mesenchymal subtype, such as *LAPTM5*. Nevertheless, only 10/33 genes in our signature were shared with the signatures used to predict immune cell types and GBM subtypes. In this manner, our work provides a different set of genes that can be helpful for the analysis of novel features of gliomas.

From the markers identified in this study, we observed that high protein levels of LAPTM5 associate with worse survival in GBMs. LAPTM5 is a transmembrane receptor associated with lysosomes and can function as a modulator of the immune system. It has been proposed that LAPTM5 downregulates the expression of T‐ and B‐cell receptors at the plasma membrane (Ouchida *et al.*, 2010; Ouchida *et al.*, 2008). In that manner, LAPTM5 might be a mechanism used by gliomas to negatively modulate the antitumoral response of the immune system. LAPTM5 has also been shown to be a proinflammatory regulator of macrophages (Glowacka *et al.*, 2012). However, a recent report suggested that LAPTM5 is downregulated in human cancers, such as neuroblastoma, and that low levels of LAPTM5 are associated with poor prognosis (Nuylan *et al.*, 2016). Our data suggest that LAPTM5 in GBMs show an opposite regulation to what has been observed in other tumor types.

We did not find differences in RSK2 expression between NB and gliomas I‐IV. This finding disagrees with a previous report claiming that GBMs express more RSK2 protein than normal brain (Mathew *et al.*, 2015). However, our conclusions are based on the expression levels of 189 samples, providing stronger support than the previous report that included two images, one for NB and one for GBM, with no further analysis. In the GBMs from our study, RSK2 protein levels were associated with worse survival and this might be related to its proposed role in migration/invasion in GBM‐derived cell lines (Sulzmaier *et al.*, 2016). It is important to note that about half of the RSK2^hi^ GBMs were also RSK1^hi^, which raises the possibility that high levels of RSK1 can define a different subset of GBMs, probably showing immune infiltration, even within the RSK2^hi^ group.

We previously showed that RSK3 and RSK4 isoforms are not detectable in two GBM‐derived cell lines (Roffé *et al.*, 2015). Accordingly, we did not detect RSK4 isoform in glioma samples. RSK3 protein expression was low in gliomas, and the higher levels were found in normal brain. In fact, GBMs show the lowest expression of RSK3. Interestingly, it has been suggested that both RSK3 and RSK4 can have tumor suppressor roles (Romeo *et al.*, 2012). TCGA RPPA data also included an antibody that detects RSK1/2/3, and our analysis indicated that GBMs show reduced RSK1/2/3 levels when compared to LGG (Fig. S12A). One possibility is that RSK3 might be present in LGG and reduced in GBMs as observed with the RSK3 antibody, but without knowing the real contribution of each individual isoform for the reactivity of RSK1/2/3 antibody is impossible to derive any conclusions. Unlike RSK1 and RSK2, the RSK1/2/3 antibody showed no apparent relationship with survival in GBMs (Fig. S12B).

The levels of P(S380)‐RSK were higher in RSK1^hi^ and, to a lesser extent, in RSK2^hi^ GBMs; however, the samples showing the highest phosphorylation levels were those bearing high RSK1 and RSK2 expression at the same time. This observation could be related to the proposed cross‐reactivity of the P(S380)‐RSK with P(S386)‐RSK2, which precludes the determination of RSK activation at the isoform level. Analysis of the RPPA data for P(T359/S363)‐RSK1 further confirmed that, in GBMs, RSK1 increases not only in expression but also in activity. In any case, our results point to the necessity of analyzing the RSK family at an isoform‐specific level.

Our results have important implications that can eventually be translated to a clinical setting. RSK1 was the only RSK isoform that increased along with the grade of the gliomas and after recurrence. This strongly suggests that RSK1 levels and activity increase during glioma progression and might be involved in this process. Since the increase in RSK1 is accompanied by high levels of immune infiltration, its kinase activity might play a role in immune system modulation for GBM and LGG. Thus, RSK1 inhibition represents a promising goal for drug development in targeted therapies. Moreover, RSK1 protein expression can be used as a molecular marker to define a group of patients that almost certainly will have a short survival. Since RSK1 appears to be enriched in GBM but not NB, an appropriate NB control could be used as a reference for IHC reactions. Moreover, the RSK1 signature can be used not only in GBM but also in LGG to predict cases with poor survival. While RSK2 is expressed at comparable levels in NB and gliomas I to IV, gain of RSK1 is a feature that was only observed in GBMs. From a therapeutic point of view, RSK1‐specific inhibition may prove useful for targeting only GBM cells and not nontumoral cells in RSK1^hi^ GBMs.

## 5 Conclusions

This study demonstrates that RSK1, but not RSK2 or RSK3, expression and RSK1 activation increase during glioma progression. Remarkably, long and very long survivors are essentially absent from the RSK1^hi^ group. Although RSK2 protein expression do not increase during glioma progression, high RSK2 levels are associated with worse survival and RSK2 behaves as an independent prognostic marker when adjusted for IDH1 mutation status and treatment. Transcriptome analysis indicated that RSK1^hi^ GBMs express mesenchymal subtype and immune infiltrate genes, showing enrichment for activated NK and M2 macrophage genes, as well as high levels of CD68 protein. An mRNA‐based RSK1 signature could be used to infer RSK1 protein levels in other glioma datasets. GBMs show enrichment in the RSK1 signature when compared to NB or LGG, further confirming the hypothesis that RSK1 expression might be gained during glioma progression together with immune infiltration. The RSK1 signature was related to worse survival in both GBMs and LGG. Thus, our results provide clinical and molecular basis to consider the kinase RSK1 as a promising biomarker and therapeutic target in gliomas.

## Conflict of interest

The authors declare no conflict of interest.

## Author contributions

MR and GNH conceived and designed the experiments. MR and GNH analyzed the data. MR and FCSL prepared the RNA. MR performed the microarrays. FF and HMB performed IHC reactions. BB and GNH performed Opal multiplex IHC experiments. MDB performed pathological anatomy analysis. CNCS, IVF, JRC, and LCT collected Recife’s cohort. PIS collected gliomas at the ACCCC for the western blot experiments. MR, GNH, and VRM wrote the manuscript.

## Supporting information


**Fig S1.** Expression of RSK isoforms in GBMs of the ACCCC cohort.

**Fig S2.** Western blot for RSK isoforms in gliomas.

**Fig S3.** Expression of RSK isoforms in GBMs and its relationship with survival and IDH1 mutation status.

**Fig S4.** Multivariate analysis for RSK2 in GBMs of the ACCCC cohort.

**Fig S5.** Analysis of RSK phosphorylation in GBMs of the ACCCC cohort.

**Fig S6.** Survival curves for the 30 GBM cases from the ACCCC cohort used for transcriptome.

**Fig S7.** Immune‐cell composition of RSK1^hi^ and RSK1^lo^ GBMs.

**Fig S8.** Expression of RSK1, LAPTM5 and CD68 in GBMs of the Recife cohort.

**Fig S9.** RSK1 relationship with prognostic markers in GBMs of the Recife cohort.

**Fig S10.** RSK1 and CD68 expression in cells of GBM tissue.

**Fig S11.** IDH1 mutation and G‐CIMP status in RSK1 signature‐enriched GBMs.

**Fig S12.** Analysis of reverse phase protein array (RPPA) data (TCGA) for RSK1/2/3 antibody in LGGs and GBM.

**Table S1.** Clinical information of cohorts used in the present study (excel file).

**Table S2.** Median survival information for the overall‐survival plots in the article.

**Table S3.** DEGs between RSK1^hi^ and RSK1^lo^ GBMs (excel file).

**Table S4.** Complete list of biological processes obtained by the GOstats package (excel file).

**Appendix S1.** Additional information of r packages used in this article.

## Data Availability

The HTA 2.0 microarray data from this study were deposited in the NCBI Database of GEO with accession number http://www.ncbi.nlm.nih.gov/geo/query/acc.cgi?acc=GSE139380.
