# Supplementary figures and images for: Aberrant expression of RSK1 characterizes high‐grade gliomas with immune infiltration
Source: Mol Oncol. 2019 Dec 11;14(1):159–79. doi: 10.1002/1878-0261.12595 (PMC6944115; doi:10.1002/1878-0261.12595)

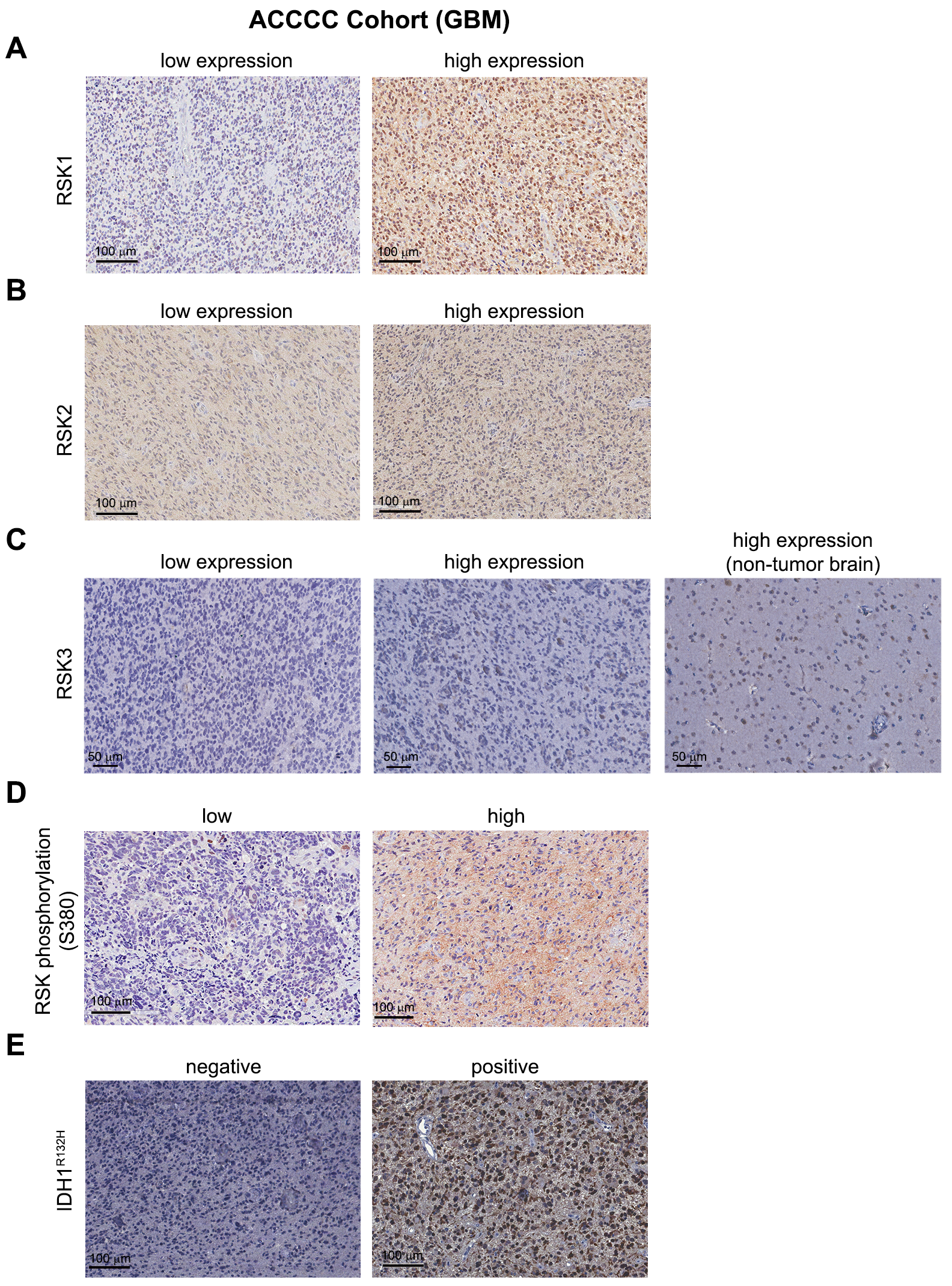

Supplement: Supplementary file 1 — Fig S1. Expression of RSK isoforms in GBMs of the ACCCC cohort. [file MOL2-14-159-s001.tif]

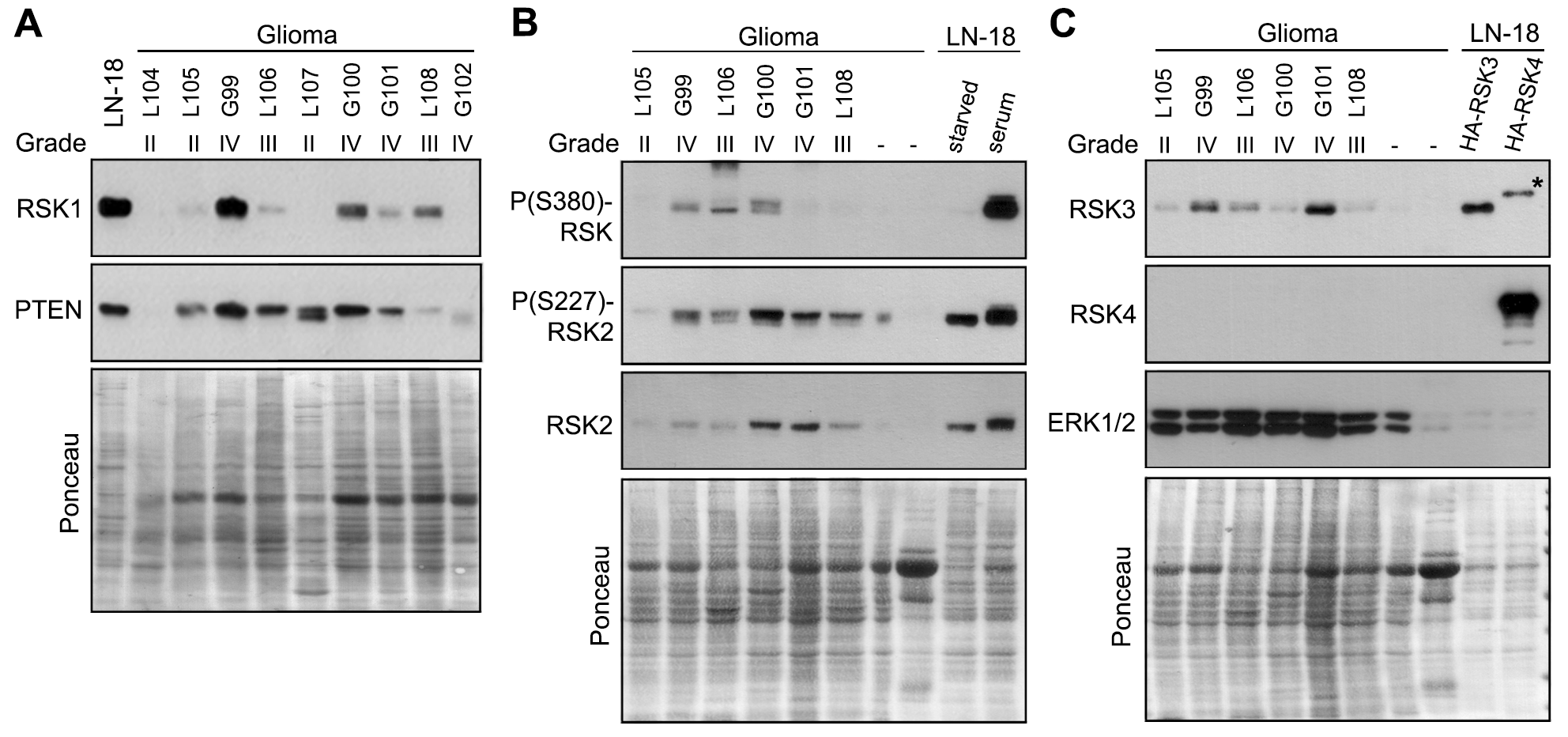

Supplement: Supplementary file 2 — Fig S2. Western blot for RSK isoforms in gliomas. [file MOL2-14-159-s002.tif]

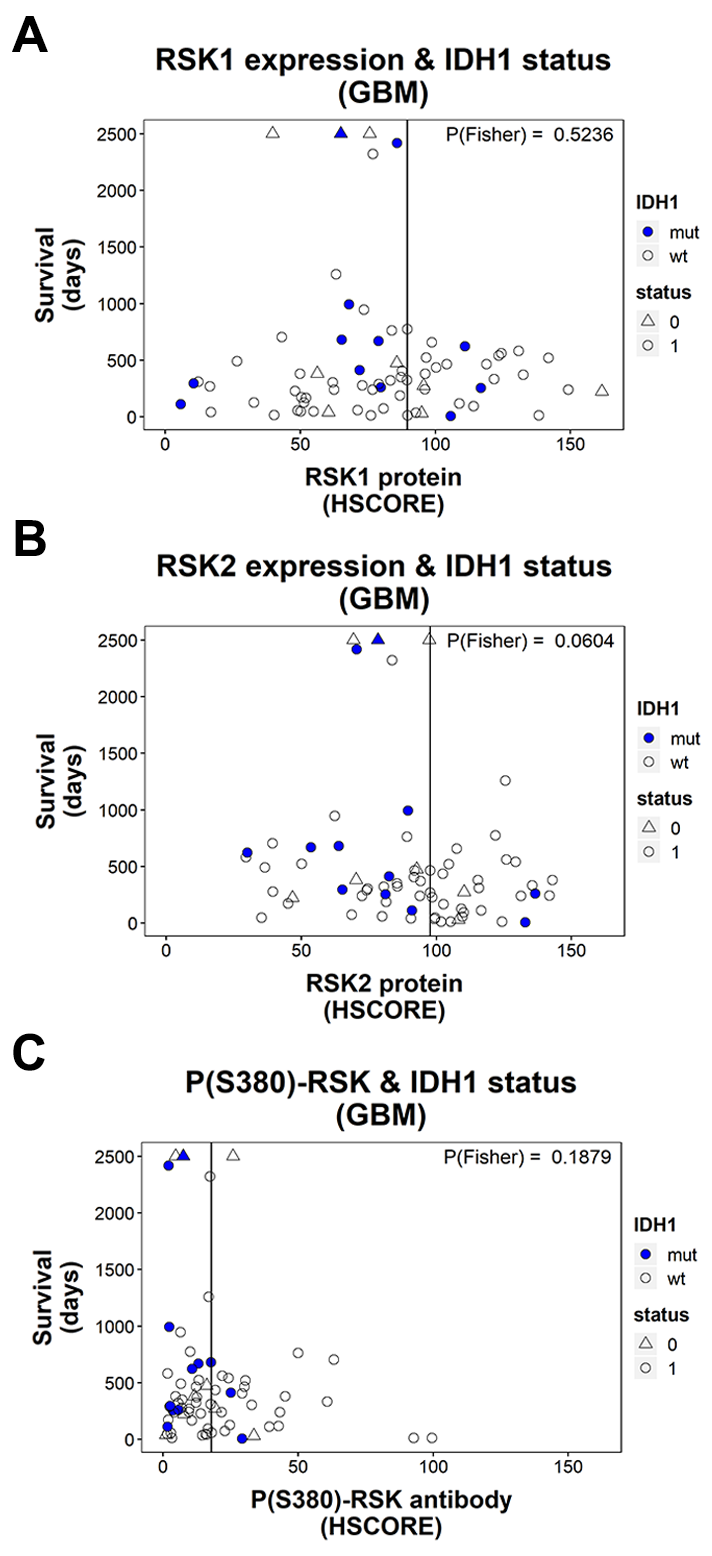

Supplement: Supplementary file 3 — Fig S3. Expression of RSK isoforms in GBMs and its relationship with survival and IDH1 mutation status. [file MOL2-14-159-s003.tif]

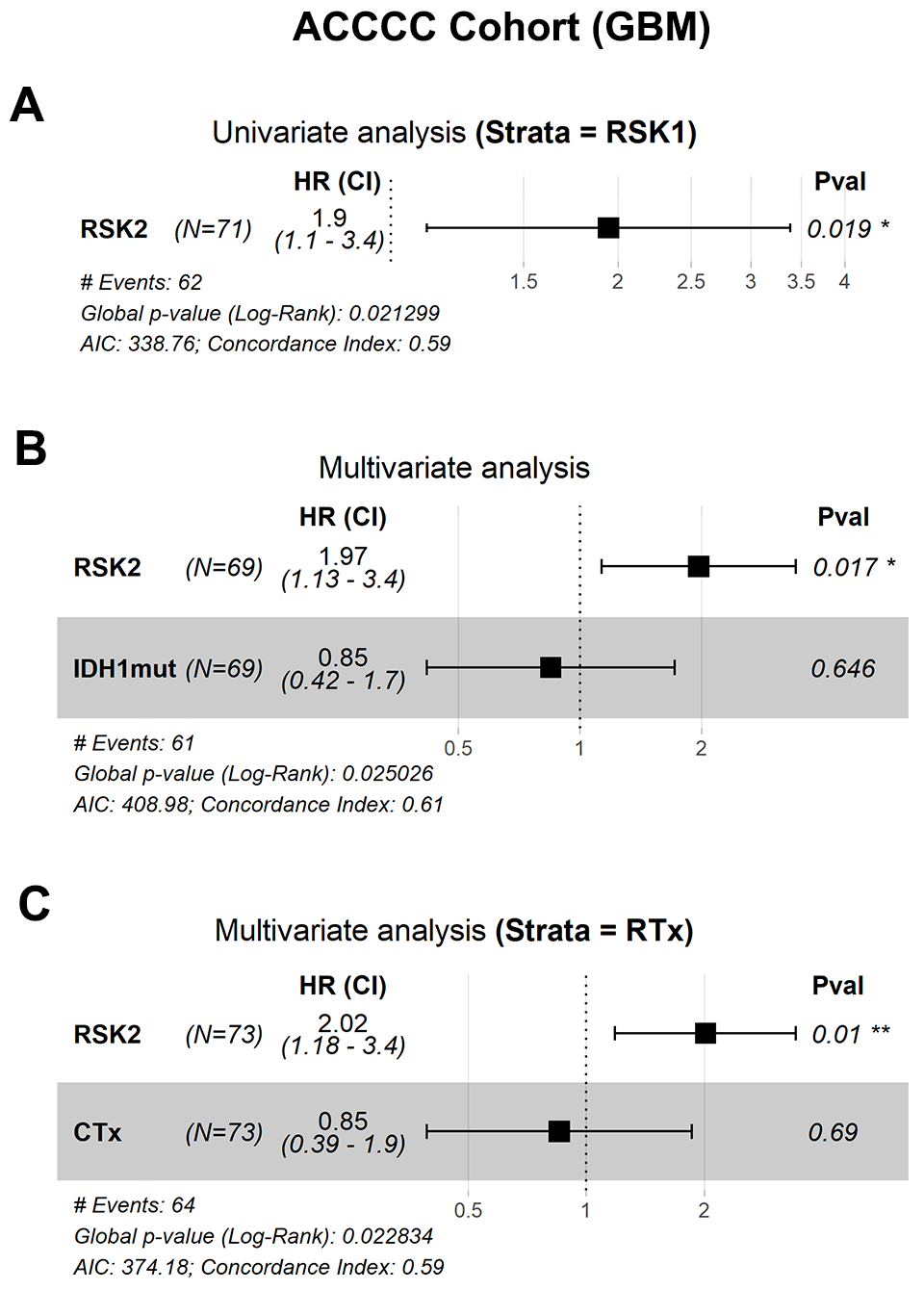

Supplement: Supplementary file 4 — Fig S4. Multivariate analysis for RSK2 in GBMs of the ACCCC cohort. [file MOL2-14-159-s004.tif]

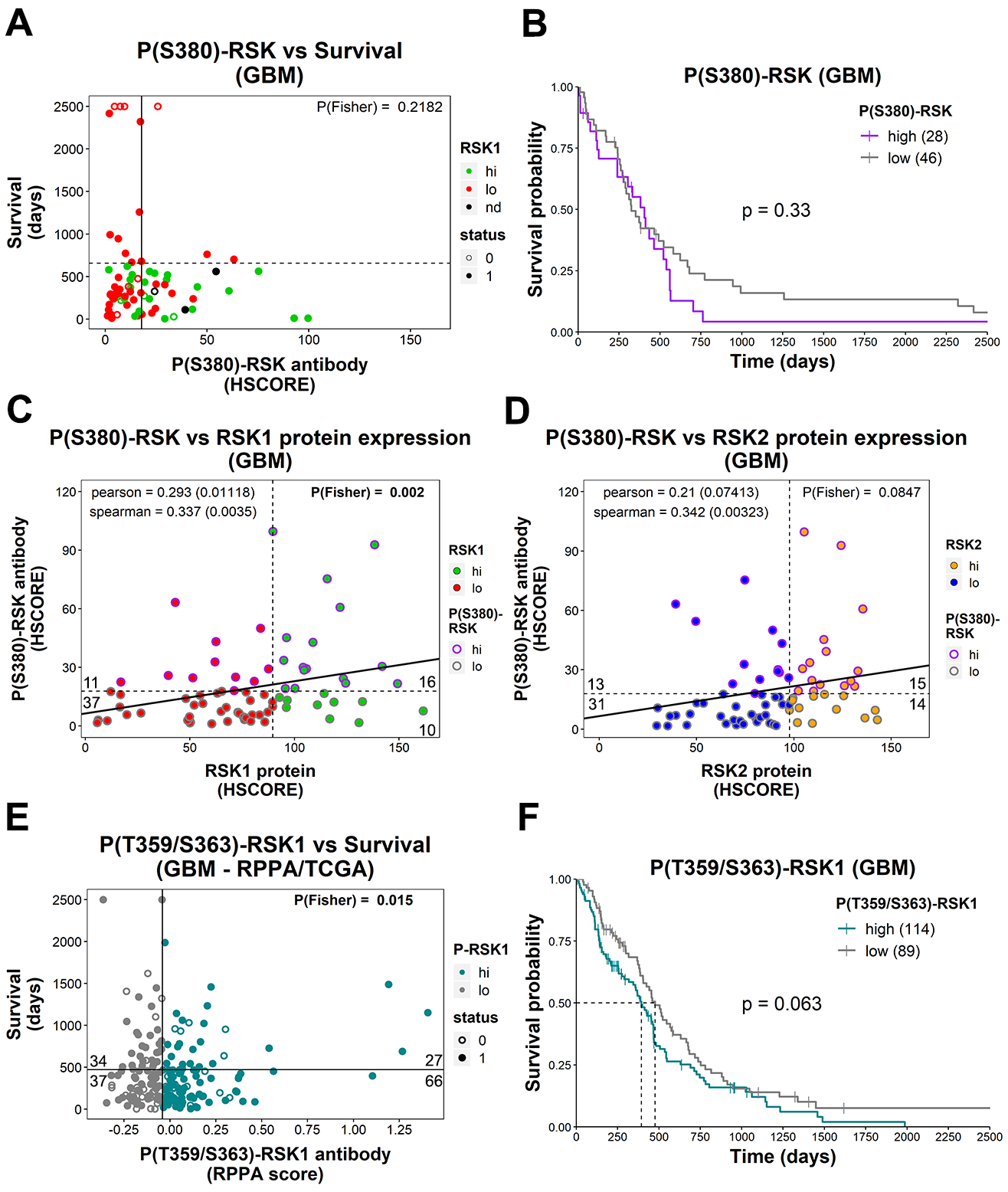

Supplement: Supplementary file 5 — Fig S5. Analysis of RSK phosphorylation in GBMs of the ACCCC cohort. [file MOL2-14-159-s005.tif]

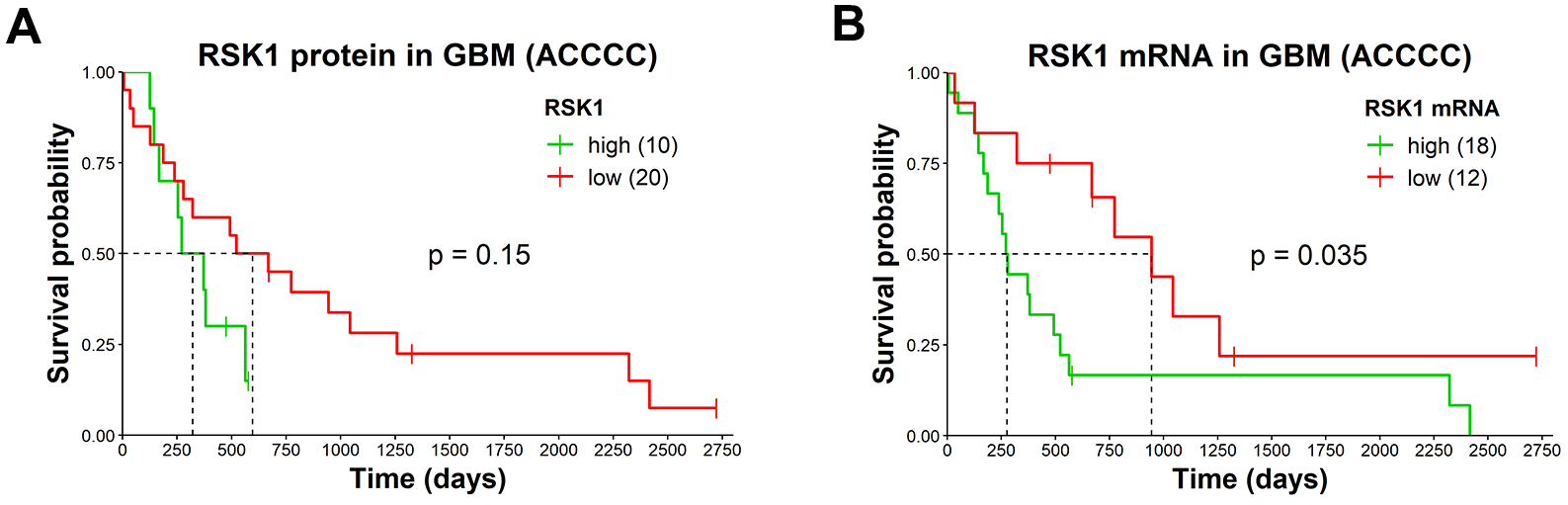

Supplement: Supplementary file 6 — Fig S6. Survival curves for the 30 GBM cases from the ACCCC cohort used for transcriptome. [file MOL2-14-159-s006.tif]

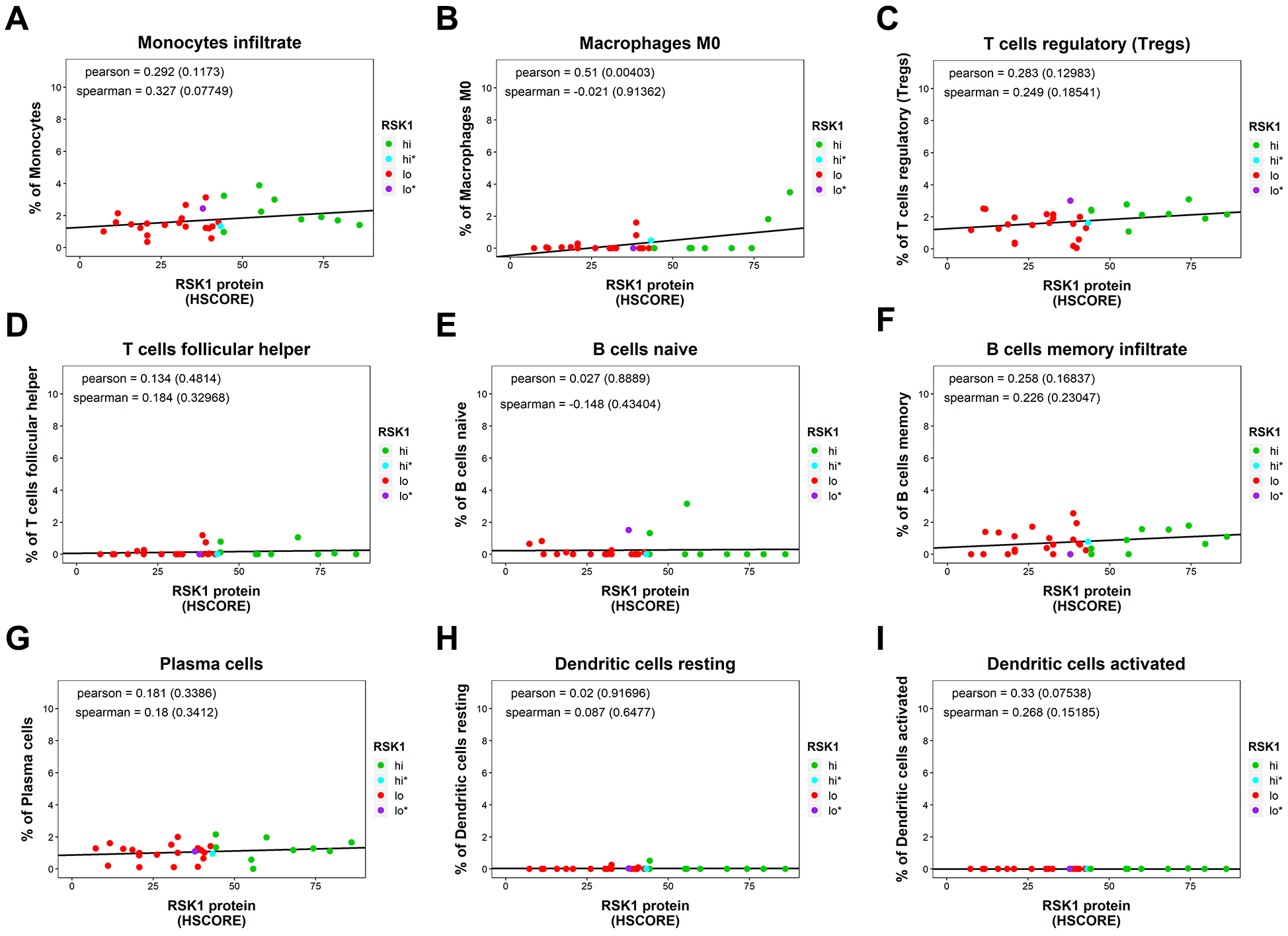

Supplement: Supplementary file 7 — Fig S7. Immune‐cell composition of RSK1hi and RSK1lo GBMs. [file MOL2-14-159-s007.tif]

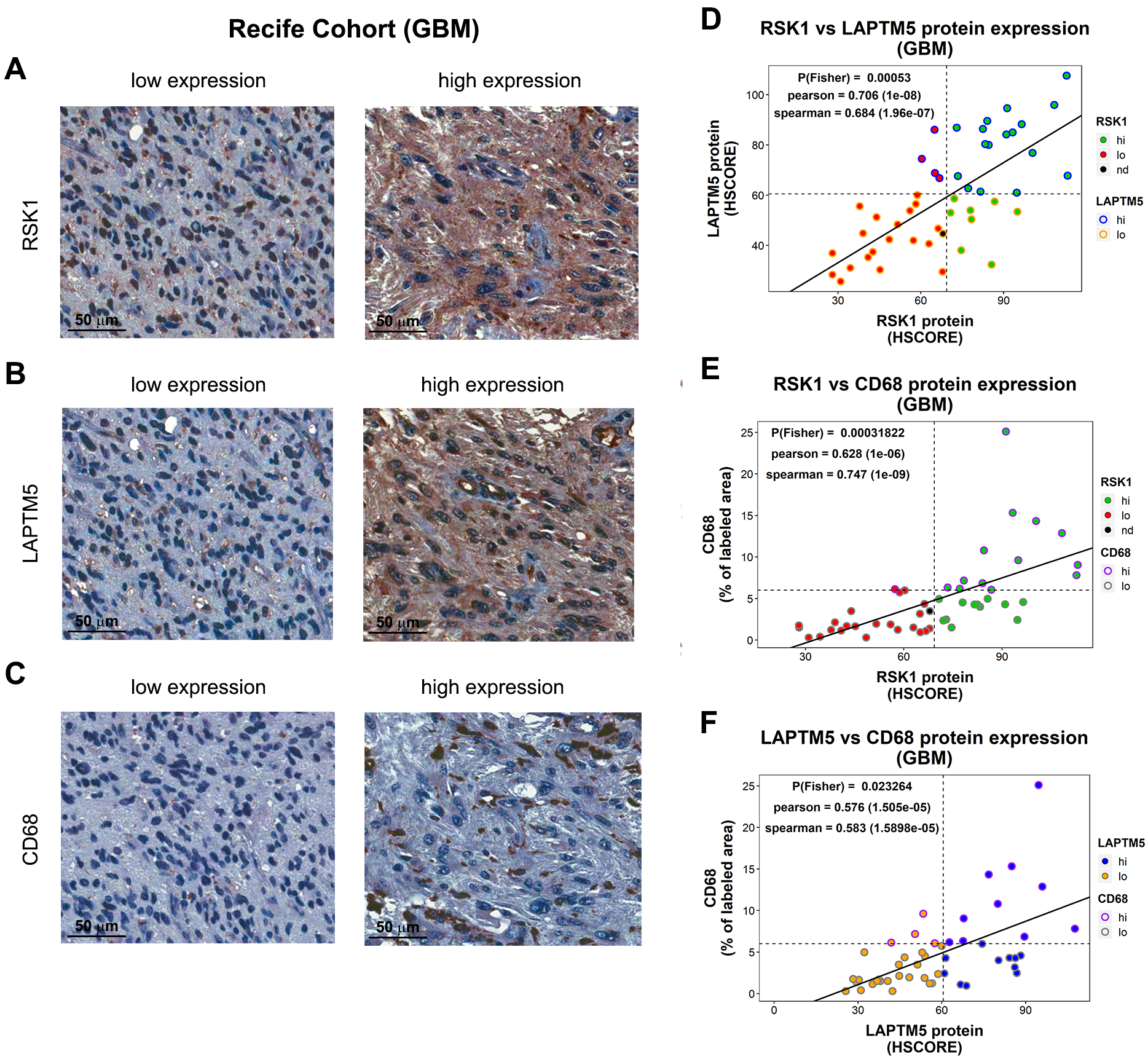

Supplement: Supplementary file 8 — Fig S8. Expression of RSK1, LAPTM5 and CD68 in GBMs of the Recife cohort. [file MOL2-14-159-s008.tif]

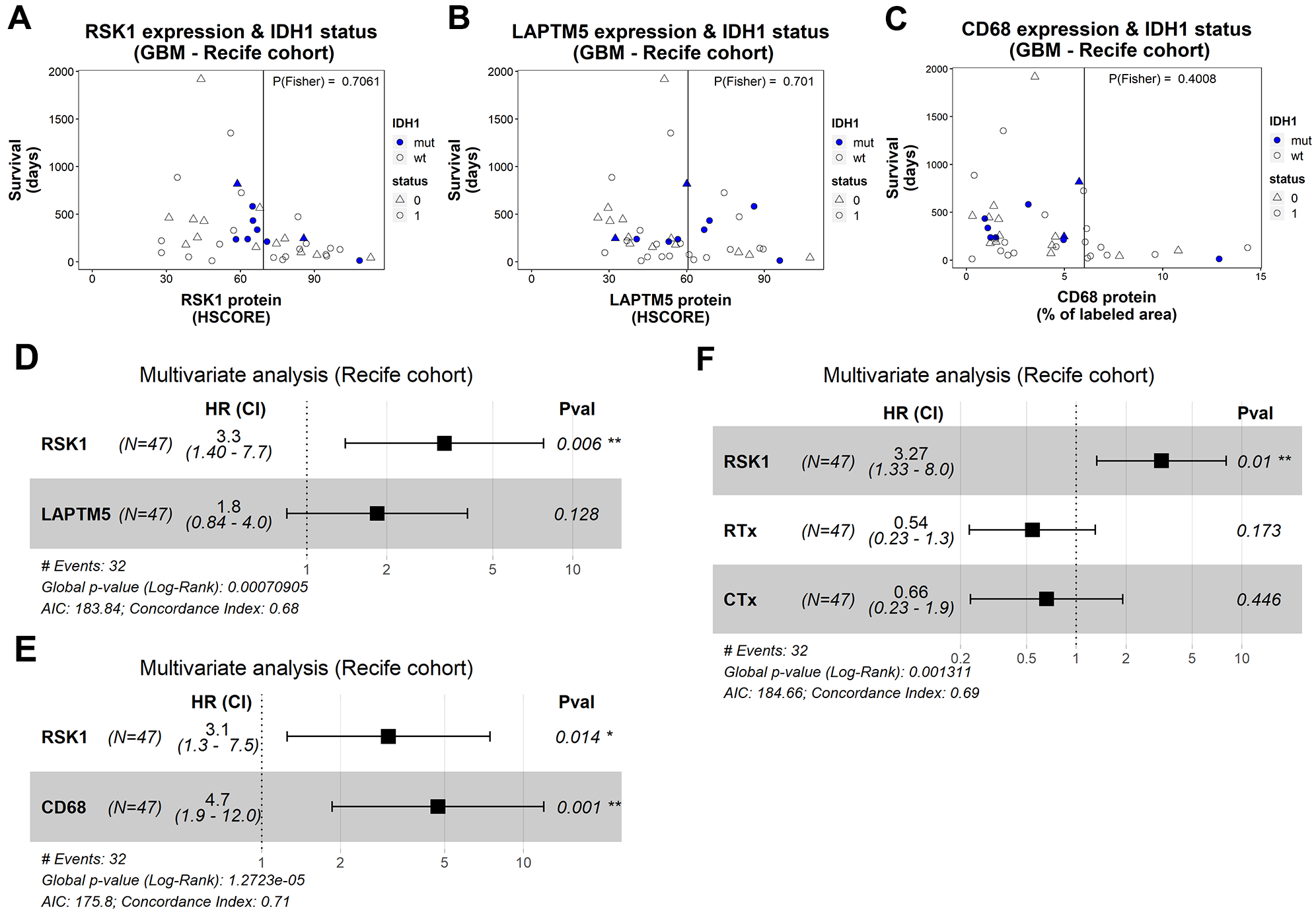

Supplement: Supplementary file 9 — Fig S9. RSK1 relationship with prognostic markers in GBMs of the Recife cohort. [file MOL2-14-159-s009.tif]

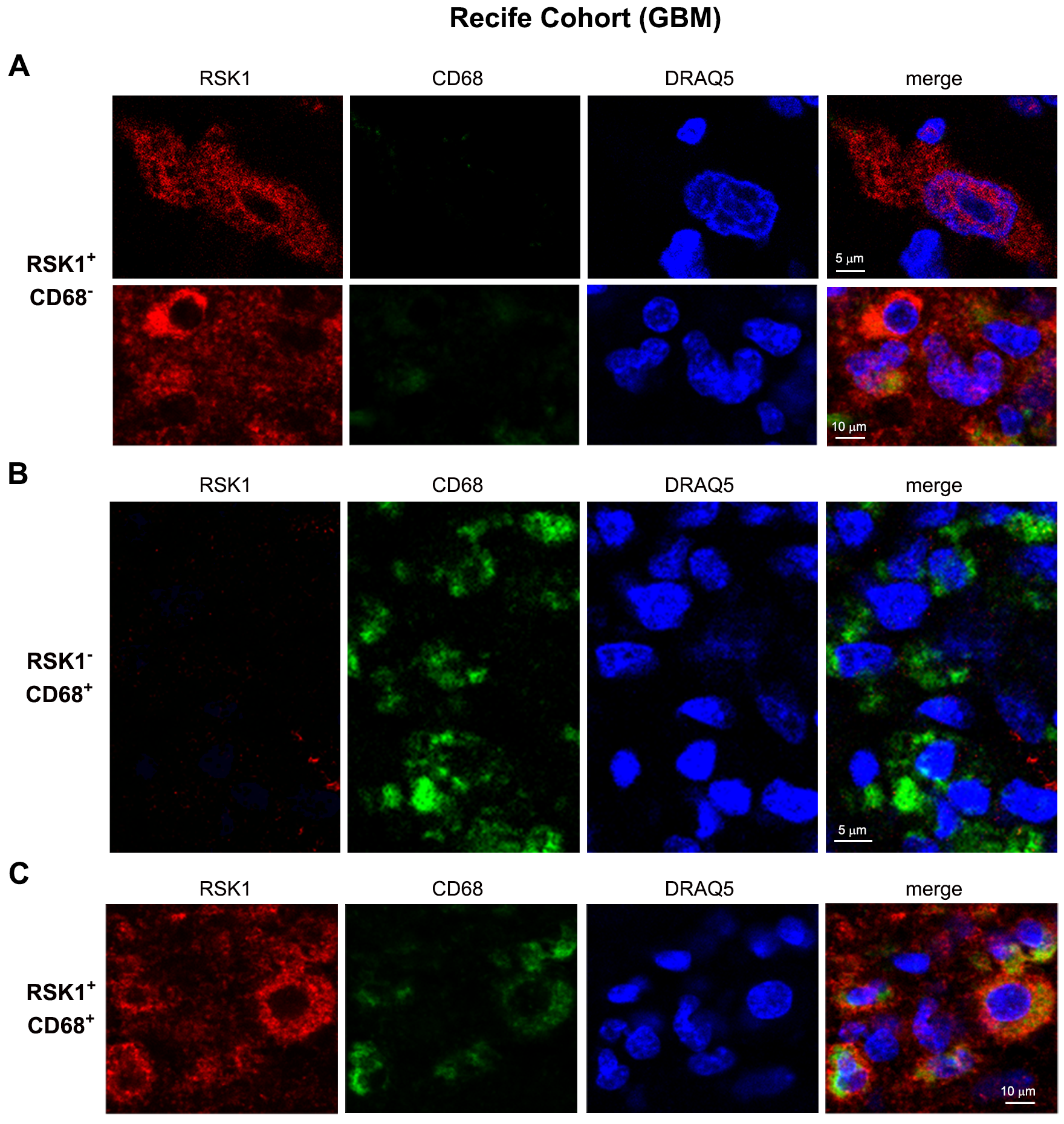

Supplement: Supplementary file 10 — Fig S10. RSK1 and CD68 expression in cells of GBM tissue. [file MOL2-14-159-s010.tif]

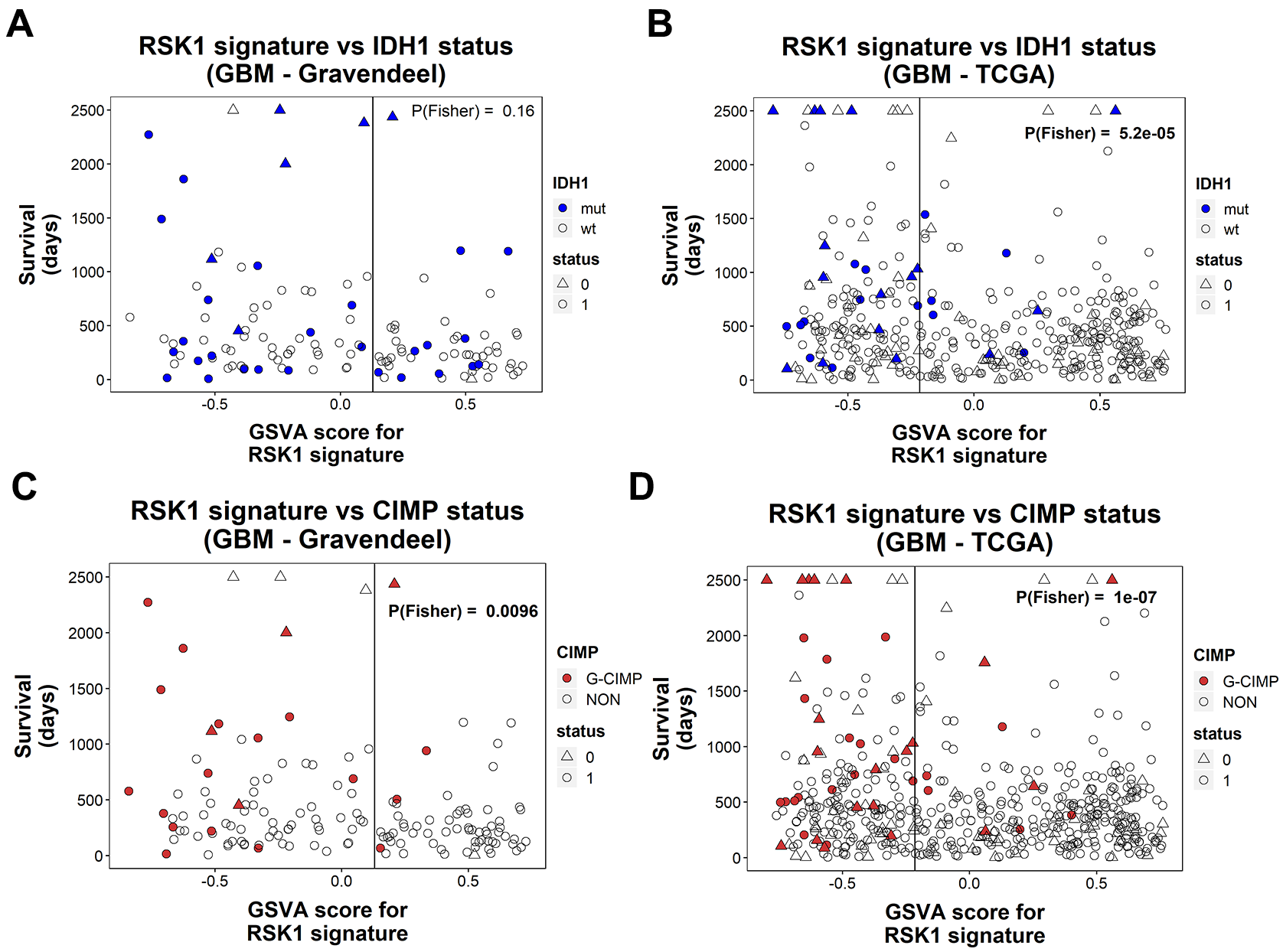

Supplement: Supplementary file 11 — Fig S11. IDH1 mutation and G‐CIMP status in RSK1 signature‐enriched GBMs. [file MOL2-14-159-s011.tif]

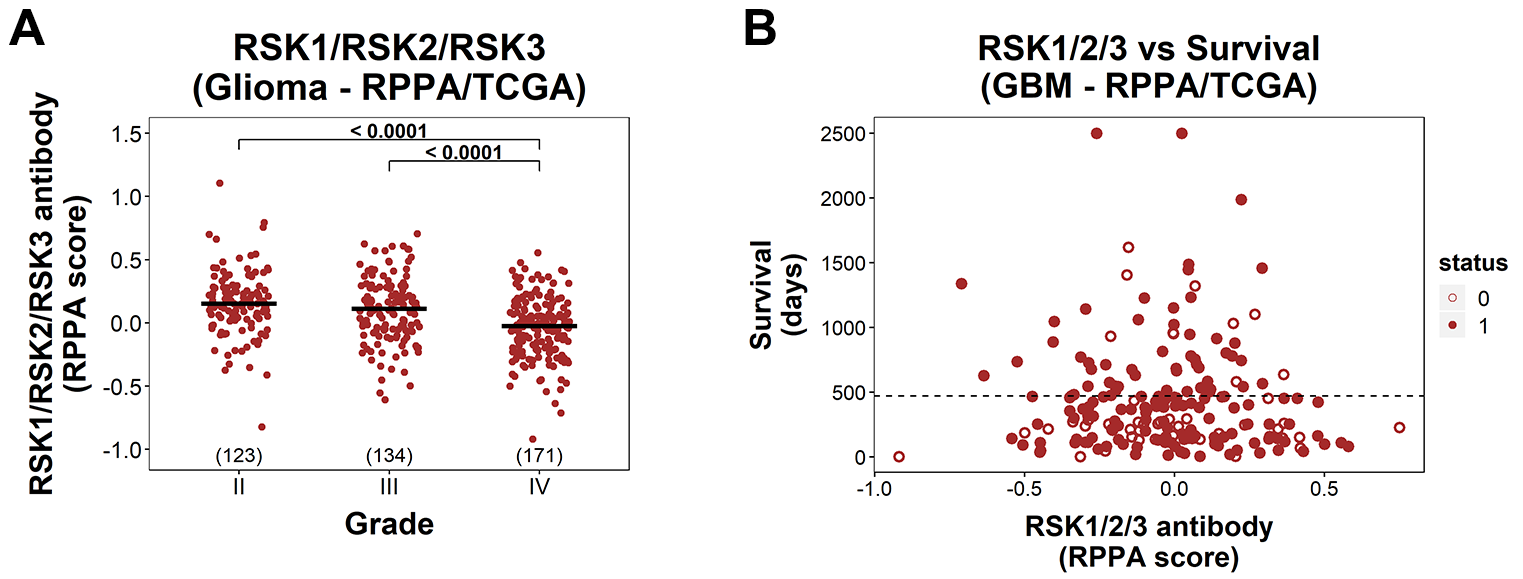

Supplement: Supplementary file 12 — Fig S12. Analysis of reverse phase protein array (RPPA) data (TCGA) for RSK1/2/3 antibody in LGGs and GBM. [file MOL2-14-159-s012.tif]
