# Supplementary material for: Aberrant expression of RSK1 characterizes high‐grade gliomas with immune infiltration
Source: Mol Oncol. 2019 Dec 11;14(1):159–79. doi: 10.1002/1878-0261.12595 (PMC6944115; doi:10.1002/1878-0261.12595)
Supplement: Supplementary file 14 — Table S2. Median survival information for the overall‐survival plots in the article. [file MOL2-14-159-s014.docx]

**Table S2.** Median survival information for the overall-survival plots in the article.

| **Figure** | **Groups** | **Survival (days)** | **N^o^ of samples** | **P (log-rank)** |
| --- | --- | --- | --- | --- |
| 2D | RSK2^hi^ | 260 | 29 | 0.0038 |
|  | RSK2^lo^ | 412 | 44 |  |
| 5B | RSK1^hi^ | 135 | 24 | 0.00032 |
|  | RSK1^lo^ | 583 | 23 |  |
| 5D | LAPTM5^hi^ | 135 | 20 | 0.0074 |
|  | LAPTM5^lo^ | 330 | 27 |  |
| 5F | CD68^hi^ | 78 | 14 | < 0.0001 |
|  | CD68^lo^ | 434 | 33 |  |
| 6C | signRSK1^enriched^ | 272 | 15 | 0.0044 |
|  | signRSK1^underrepresented^ | 945 | 15 |  |
| 6H | signRSK1^enriched^ | 211.5 | 67 | 0.00069 |
|  | signRSK1^underrepresented^ | 339 | 81 |  |
| 6I | signRSK1^enriched^ | 382 | 320 | < 0.0001 |
|  | signRSK1^underrepresented^ | 480 | 202 |  |
| 7E | signRSK1^enriched^ | 1033 | 67 | 0.0031 |
|  | signRSK1^underrepresented^ | 1762 | 169 |  |
| 7F | signRSK1^enriched^ | 552 | 35 | 0.00099 |
|  | signRSK1^underrepresented^ | 1863 | 50 |  |
| 7G | signRSK1^enriched^ | 2907 | 107 | 0.0074 |
|  | signRSK1^underrepresented^ | 5166 | 108 |  |
| S5F | P(T359/S636)-RSK1^hi^ | 394 | 114 | 0.063 |
|  | P(T359/S636)-RSK1^lo^ | 476 | 89 |  |
| S6A | RSK1^hi^ | 322 | 10 | 0.15 |
|  | RSK1^lo^ | 596 | 20 |  |
| S6B | RSK1-mRNA^hi^ | 276 | 18 | 0.035 |
|  | RSK1-mRNA^lo^ | 945 | 12 |  |
