# Supplementary material for: Aberrant expression of RSK1 characterizes high‐grade gliomas with immune infiltration
Source: Mol Oncol. 2019 Dec 11;14(1):159–79. doi: 10.1002/1878-0261.12595 (PMC6944115; doi:10.1002/1878-0261.12595)
Supplement: Supplementary file 17 — Appendix S1. Additional information of r packages used in this article. [file MOL2-14-159-s017.docx]

**Additional information of R packages used in this article**

1. Bengtsson H, Bullard J, Hansen K (2018). *affxparser: Affymetrix File Parsing SDK*. R package version 1.54.0, <https://github.com/HenrikBengtsson/affxparser>.

2. MacDonald JW (2017). hta20transcriptcluster.db: Affymetrix hta20 annotation data (chip hta20transcriptcluster). R package version 8.7.0.

3. H. Wickham. ggplot2: Elegant Graphics for Data Analysis. Springer-Verlag New York, 2016.

4. herneau T (2015). A Package for Survival Analysis in S. version 2.38, [https://CRAN.R-project.org/package=survival](https://cran.r-project.org/package=survival).

5. Carlson M (2018). org.Hs.eg.db: Genome wide annotation for Human. R package version 3.7.0

6. Carlson M (2016). hthgu133a.db: Affymetrix HT Human Genome U133 Array Plate Set annotation data (chip hthgu133a). R package version 3.2.3.

7. Carlson M (2016). hgu133plus2.db: Affymetrix Human Genome U133 Plus 2.0 Array annotation data (chip hgu133plus2). R package version 3.2.3.
