# Supplementary material for: Aberrant expression of RSK1 characterizes high‐grade gliomas with immune infiltration
Source: Mol Oncol. 2019 Dec 11;14(1):159–79. doi: 10.1002/1878-0261.12595 (PMC6944115; doi:10.1002/1878-0261.12595)
Supplement: Supplementary file 18 [file MOL2-14-159-s018.docx]

**Supplemental figure legends**

**Fig. S1.** Expression of RSK isoforms in glioblastomas of the ACCCC cohort. Immunohistochemistry reactions for (A) RSK1, (B) RSK2, (C) RSK3, (D) P(S380)-RSK and (E) IDH1^R132H^. Representative images of high and low expression in GBMs are shown. For RSK3, we included an image of a non-tumor brain showing high RSK3 levels. Scale bars: 100 μm (for RSK3: 50 μm).

**Fig. S2.** Western blot for RSK isoforms in gliomas. Extracts from freshly collected glioma samples of different grades were prepared and used for western blots. (A) Western blot for RSK1. Extract of LN-18 cells, which express high RSK1 levels, was included as control. Western blot for PTEN was included for comparison of the expression levels. (B) Western blot for RSK2 and P(S380)-RSK. Extracts of LN-18 cells that were serum-starved for 48 hours and treated for 15 minutes with 10% serum after starvation were included as controls for phosphorylation. Western blot for phosphorylated RSK2 (S227) was included for comparison of the phosphorylation levels. According to the manufacturer (*Cell Signaling*), this antibody can also detect RSK1 phosphorylation at S221. (C) Western blot for RSK3 and RSK4. The western blots for RSK3 and RSK4 were over-exposed due to their very low/absent protein levels. Since LN-18 cells do not express these isoforms, extracts of LN-18 cells, transfected to express HA-RSK3 or HA-RSK4, were included as controls. This membrane was initially incubated with RSK4 and then stripped and reprobed with RSK3 antibody. *band corresponding to left-over anti-RSK4 that appeared because of long exposure times. Western blot for ERK1/2 was included as a loading control. Lanes labeled with a dash (-) correspond to extracts of frozen glioma samples. The quality of these samples was low. For all the western blots, the Ponceau-stained membrane is shown as a loading control.

**Fig. S3.** Expression of RSK isoforms in glioblastomas and its relationship with survival and IDH1 mutation status. Graphs for (A) RSK1 and (B) RSK2 protein levels and (C) P(S380)-RSK levels, and their relationship with survival times, in GBMs (grade IV glioma). The vertical line indicates the cutoff for high- and low-expression groups. The P-value of the Fisher’s exact test for the presence of IDH1^R132H^ in high- and low-expression groups is indicated. Status: 1 = dead; 0 = censored.

**Fig. S4.** Multivariate analysis for RSK2 in glioblastomas of the ACCCC cohort. (A) Since it was not possible to assume proportional Hazards for RSK1, univariate Cox analysis for RSK2 with stratification by RSK1^hi^ and RSK1^lo^ groups was performed. (B) Multivariate Cox analysis with RSK2 and IDH1^R132H^ as covariates. (C) Since it was not possible to assume proportional Hazards for radiotherapy (RTx), a multivariate Cox analysis using RSK2 and chemotherapy (CTx) as covariates, was performed with stratification by RTx.

**Fig. S5.** Analysis of RSK phosphorylation in glioblastomas of the ACCCC cohort. (A) Graph for P(S380)-RSK levels relative to survival in GBMs. Colors indicate whether the samples belong to RSK1^hi^ or RSK1^lo^ groups. The survival time cutoff (horizontal line) was set to the longest survival time for RSK1^hi^ (Fig. 2A). The cutoff for high- and low-expression groups (vertical line) corresponds to the one that resulted in the minimum P-value of the Fisher’s exact test. Status: 1 = dead; 0 = censored. (B) Overall survival plot comparing P(S380)-RSK^hi^ and P(S380)-RSK^lo^ groups. The number of samples is indicated in parentheses. (C,D) Correlation between P(S380)-RSK levels and (C) RSK1 or (D) RSK2 protein expression in GBMs. The cutoff for high- and low-expression groups is indicated by a vertical line for RSK1 or RSK2 and a horizontal line for P(S380)-RSK. The number of samples in each quadrant and the P-value for the Fisher´s exact test are indicated. (E,F) Analysis of reverse phase protein array (RPPA) data (TCGA) for P(T359/S363)-RSK1 antibody in GBM. (E) P(T359/S363)-RSK1 levels and their relationship with survival times in GBM. The vertical line indicates the cutoff for P(T359/S363)-RSK1^hi^ and P(T359/S363)-RSK1^lo^ groups and the horizontal line indicates the cutoff for survival time. The P-value for the Fisher´s exact test and the number of samples in each quadrant are indicated. Censored samples below the survival time cutoff were not considered. (C) Overall survival plot comparing P(T359/S363)-RSK1^hi^ and P(T359/S363)-RSK1^lo^. The number of samples is indicated in parentheses.

**Fig. S6.** Survival curves for the 30 glioblastoma cases from the ACCCC cohort used for transcriptome. Overall survival plots comparing (A) RSK1-mRNA^hi^ and RSK1-mRNA^lo^ groups or (B) RSK1^hi^ and RSK1^lo^ groups. The number of samples is indicated in parentheses.

**Fig. S7.** Immune-cell composition of RSK1^hi^ and RSK1^lo^ glioblastomas. The transcriptome data from the 30 GBMs from the ACCCC cohort were used as input for the CIBERSORT algorithm using the LM22 signature to estimate the fraction of immune-related cells. (A-I) cell types that did not correlate with RSK1 protein levels. Signatures not present in GBM samples: T cells CD4 naive, T cells CD4 memory activated, and T cells gamma delta.

**Fig. S8.** Expression of RSK1, LAPTM5 and CD68 in glioblastomas of the Recife cohort. Immunohistochemistry reactions for (A) RSK1, (B) LAPTM5 and (C) CD68. Representative images are shown. Scale bars: 50 μm. (D-F) Graphs showing the relationship among (D) RSK1 and LAPTM5, (E) RSK1 and CD68, and (F) LAPTM5 and CD68. The cutoff for high- and low-expression groups is indicated by vertical and horizontal lines. The P-value for the Fisher´s exact test is indicated.

**Fig. S9.** RSK1 relationship with prognostic markers in glioblastomas of the Recife cohort. IDH1^R132H^ status in GBMs (grade IV glioma) is indicated in graphs for (A) RSK1, (B) LAPTM5 and (C) CD68 protein levels vs. survival times. The vertical line indicates the cutoff for high- and low-expression groups. The P-value of the Fisher’s exact test for the presence of IDH1^R132H^ in high- and low-expression groups is indicated. Status: 1 = dead; 0 = censored. (D,E) Multivariate Cox analysis for RSK1 with (D) LAPTM5 or (E) CD68 as covariates. (F) Multivariate Cox analysis with RSK1, radiotherapy (RTx) and chemotherapy (CTx) as covariates.

**Fig. S10.** RSK1 and CD68 expression in cells of glioblastoma tissue. Multiplex immunohistofluorescence detection of RSK1, CD68, and nuclei labeling (DRAQ5) in RSK^hi^ GBM tissue. (A) RSK1^+^ CD68^-^ cells. Scale bars: 5 μm for top panels and 10 μm for bottom panels. (B) RSK1^-^ CD68^+^ cells. Scale bars: 5 μm. (C) RSK1^+^ CD68^+^ cells. Scale bars: 10 μm.

**Fig. S11.** IDH1 mutation and G-CIMP status in RSK1 signature-enriched GBMs. RSK1 signature enrichment (GSVA score) was plotted relative to patient survival for (A) Gravendeel and (B) TCGA datasets. IDH1^R132H^ status is indicated in blue. Cutoff for signRSK1^enriched^ and signRSK1^underrepresented^ groups is indicated by a vertical line. P-values for the Fisher’s exact test for the presence of IDH mutation when groups are defined according to the enrichment in RSK1 signature (GSVA score) is indicated. It is important to note that IDH1 mutant cases are infrequent in the TCGA cohort compared with other cohorts (Brennan *et al.*, 2013). (C,D) Graphs showing the RSK1 signature enrichment and the survival times for (C) Gravendeel and (D) TCGA datasets, together with the CIMP status. Cutoff for signRSK1^enriched^ and signRSK1^underrepresented^ groups is indicated by a vertical line. P-value of the Fisher’s exact test for the presence of G-CIMP when groups are defined according to their enrichment in RSK1 signature (GSVA score) is indicated. Status: 1 = dead; 0 = censored.

**Fig. S12.** Analysis of reverse phase protein array (RPPA) data (TCGA) for RSK1/2/3 antibody in low-grade gliomas and glioblastoma. (A) RSK1/2/3 levels were compared for grade II, grade III and grade IV (GBM) cases. The number of samples for each grade is indicated in parentheses. (B) RSK1/2/3 levels and their relationship with survival time in GBM. The horizontal line indicates the cutoff for survival time from Figure S5E as a reference. Status: 1 = dead; 0 = censored.
