# Supplementary material for: Aberrant expression of RSK1 characterizes high‐grade gliomas with immune infiltration
Source: Mol Oncol. 2019 Dec 11;14(1):159–79. doi: 10.1002/1878-0261.12595 (PMC6944115; doi:10.1002/1878-0261.12595)
Supplement: Supplementary file 19 [file MOL2-14-159-s019.docx]

**Supplemental table legends**

**Table S1.** Clinical information of cohorts used in the present study (excel file). Tab 1 – ACCCC cohort of astrocytomas used for TMA. Tab 2 – 30 samples of GBMs from the ACCCC cohort used for the transcriptome analysis. The HSCORE for RSK1 was obtained from the immunohistochemistry of complete tissue slices and not from a TMA spot. Thus, HSCORE might differ from the one described in Tab 1. Samples in bold were used both for TMA and microarray. Tab 3 – Glioma samples from the ACCCC used for western blot analysis. Tab 4 – Recife cohort.

**Table S2.** Median survival information for the overall-survival plots in the article.

**Table S3.** Differentially expressed genes (DEGs) between RSK1^hi^ and RSK1^lo^ glioblastomas (excel file). Tab 1 - *limma* package was used to compare RSK1^hi^ and RSK1^lo^ samples (Fig. 3A). RSK1^hi*^ and RSK1^lo*^ samples were not included in this analysis. The table represents the output. Positive logFC (log fold-change) refers to mRNAs expressed at higher levels in the RSK1^hi^ group. Tab 2 – Output of the second round of *limma* analysis using the DEGs from Tab 1 as input. The comparison was performed between RSK1^hi^ group including the RSK1^hi*^ sample and the RSK1^lo^ group including the RSK1^lo*^ sample. The mRNAs composing the RSK1 signature are indicated in green.

**Table S4.** Complete list of biological processes obtained by the GOstats package (excel file). The first tab shows the processes enriched in RSK1^hi^ GBMs and the second tab shows the processes enriched in RSK1^lo^ GBMs. The most enriched processes were used for Figure 4A.
